# AXL as immune regulator and therapeutic target in Acute Myeloid Leukemia: from current progress to novel strategies

**DOI:** 10.1186/s40164-024-00566-8

**Published:** 2024-10-04

**Authors:** Niels Vandewalle, Nathan De Beule, Ann De Becker, Elke De Bruyne, Eline Menu, Karin Vanderkerken, Karine Breckpot, Nick Devoogdt, Kim De Veirman

**Affiliations:** 1 Translational Oncology Research Center (TORC), Team Hematology and Immunology (HEIM), Vrije Universiteit Brussel (VUB), Laarbeeklaan 103, Brussels, 1090 Belgium; 2 Translational Oncology Research Center (TORC), Team Hematology and Immunology (HEIM), Hematology Department, Vrije Universiteit Brussel (VUB), Universitair Ziekenhuis Brussel (UZ Brussel), Laarbeeklaan 101, Brussels, 1090 Belgium; 3 Translational Oncology Research Center (TORC), Team Laboratory of Cellular and Molecular Therapy (LMCT), Vrije Universiteit Brussel (VUB), Laarbeeklaan 103, Brussels, 1090 Belgium; 4 Laboratory of Molecular Imaging and Therapy (MITH), Vrije Universiteit Brussel (VUB), Laarbeeklaan 103, Brussels, 1090 Belgium

**Keywords:** AXL, Immunoregulation, Therapy, Acute Myeloid Leukemia

## Abstract

Until recently, treatment options for patients diagnosed with Acute Myeloid Leukemia (AML) were limited and predominantly relied on various combinations, dosages, or schedules of traditional chemotherapeutic agents. Patients with advanced age, relapsed/refractory disease or comorbidities were often left without effective treatment options. Novel advances in the understanding of leukemogenesis at the molecular and genetic levels, alongside recent progress in drug development, have resulted in the emergence of novel therapeutic agents and strategies for AML patients. Among these innovations, the receptor tyrosine kinase AXL has been established as a promising therapeutic target for AML. AXL is a key regulator of several cellular functions, including epithelial-to-mesenchymal transition in tumor cells, immune regulation, apoptosis, angiogenesis and the development of chemoresistance. Clinical studies of AXL inhibitors, as single agents and in combination therapy, have demonstrated promising efficacy in treating AML. Additionally, novel AXL-targeted therapies, such as AXL-specific antibodies or antibody fragments, present potential solutions to overcome the limitations associated with traditional small-molecule AXL inhibitors or multikinase inhibitors. This review provides a comprehensive overview of the structure and biological functions of AXL under normal physiological conditions, including its role in immune regulation. We also summarize AXL’s involvement in cancer, with a specific emphasis on its role in the pathogenesis of AML, its contribution to immune evasion and drug resistance. Moreover, we discuss the AXL inhibitors currently undergoing (pre)clinical evaluation for the treatment of AML.

## Introduction

Acute Myeloid Leukemia (AML) is one of the most challenging hematological malignancies and is characterized by clonal expansion of myeloid blasts in the bone marrow (BM) often leading to circulating blasts in peripheral blood [1]. The uncontrolled growth of blasts leads to the suppression of normal hematopoiesis, causing BM failure and impairing the production of normal blood cells [2]. AML primarily affects older adults, with a median age at diagnosis around 68 years and a 5-year survival rate below 20% for patients between 60 and 74 years of age [3].

AML is a highly heterogeneous disease with regard to cell morphology, cytogenetics and gene mutations [4]. The prognosis of AML patients is variable and is based on clinical features (e.g., age, comorbidities and general performance) and underlying genetic features, including both cytogenetic and molecular aberrations [5]. Common genetic mutations observed in AML include mutations in genes encoding transcription factors (e.g., *RUNX1*,* CEBPA*), signaling molecules (e.g., *RAS*), epigenetic modifiers (e.g., *DNMT3A*,* IDH1/2*), and tumor suppressors (e.g., *TP53*). Approximately 20% of the AML patients harbor internal tandem duplication (ITD) mutations in the Fms-like tyrosine kinase-3 (*FLT3*) gene, which are consistently associated with a poor prognosis [6, 7]. Another common genetic alteration is the nucleophosmin 1 (*NPM1*) mutation, occurring in approximately one-third of AML cases. The *NPM1* mutation is considered a favorable prognostic marker when detected as an isolated mutation (i.e., without concurrent FLT3-ITD mutations) and is associated with higher rates of complete remission (CR) and overall survival (OS) [8].

However, despite significant advances in the understanding of AML pathogenesis, the main treatment for most AML patients remains standard chemotherapy, including cytarabine combined with an anthracycline, and the use of allogeneic hematopoietic stem cell (HSC) transplantation [9]. During the past years, the Food and Drug Administration (FDA) also approved numerous targeted therapies for AML, including FLT3 inhibitors, isocitrate dehydrogenase 1 (IDH1) inhibitors, B-cell lymphoma 2 (BCL-2) inhibitors and hypomethylating agents, allowing a more personalized treatment approach [10].

In recent years, the receptor tyrosine kinase (RTK) AXL has been identified as another critical player in AML pathogenesis. AXL was found to be overexpressed in AML patients and was associated with a poor prognosis [11, 12]. Since AXL regulates several processes involved in cancer pathogenesis, including cell survival and proliferation, angiogenesis, epithelial-to-mesenchymal transition (EMT), stem cell maintenance and immunological responses, therapeutic targeting of the AXL signaling pathway became an attractive treatment approach [13].

In this review, we discuss the function of AXL in maintaining normal physiological functions, particularly in immune regulation, and examine its involvement in cancer, with an emphasis on AML. We explore AXL’s contribution to AML pathogenesis, including mechanisms of immune evasion and drug resistance, and provide an overview of current (pre)clinical AXL inhibitors being evaluated for AML.

## AXL function in physiological conditions and cancer

### AXL structure and activation regulation

**Fig. 1:**
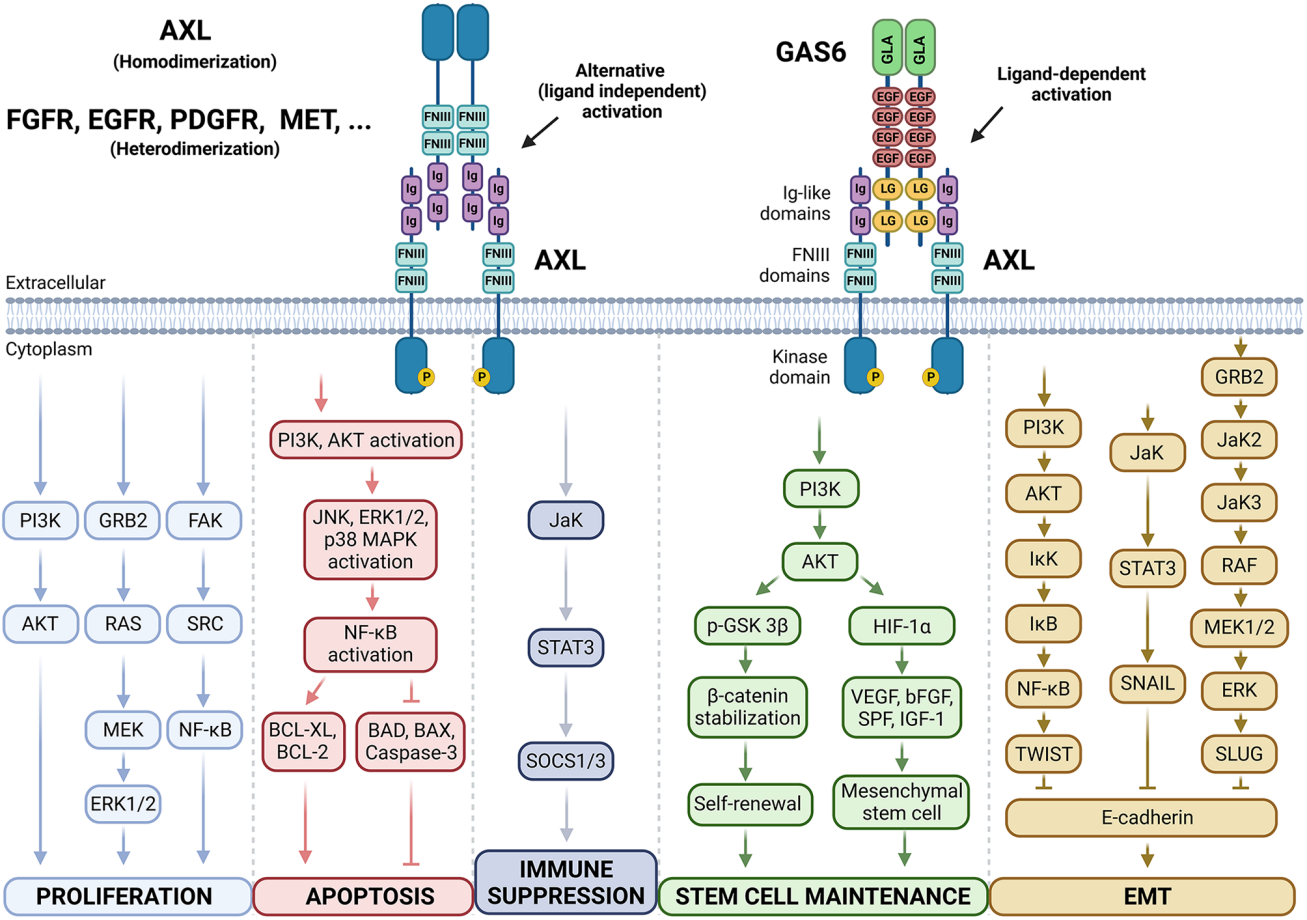
Schematic representation of the basic structure, activation and downstream signaling pathways of AXL. AXL is composed of two immunoglobulin (Ig)-like repeats and two fibronectin type III (FNIII)-like repeats, a transmembrane domain and an intracellular kinase domain. The latter can be phosphorylated through classical ligand-mediated activation upon interaction with GAS6, a vitamin K-dependent protein that binds AXL with higher affinity compared to TYRO3 or MERTK. The Gla domain of GAS6 allows for cell membrane contact and the LG domains bind the Ig-like domains of AXL. Alternatively, AXL can undergo ligand-independent activation via interaction with another AXL receptor (homodimerization), or via interaction with other receptor tyrosine kinases (heterodimerization). Upon interaction, AXL will undergo dimerization and phosphorylation, which subsequently leads to the activation of a plethora of downstream signaling cascades contributing to, among others, proliferation, apoptosis, immune suppression, stem cell maintenance or epithelial-to-mesenchymal transition (EMT). PI3K, Phosphatidylinositol-3-kinase; GRB2, Growth Factor Receptor-Bound Protein 2; FAK, Focal Adhesion Kinase; AKT, Protein Kinase B; RAS, Rat Sarcoma Virus Oncogene; SRC, Proto-Oncogene Tyrosine-Protein Kinase; MEK, Mitogen-Activated Protein Kinase Kinase; NF-κB, Nuclear Factor Kappa-Light-Chain-Enhancer of Activated B Cells; ERK1/2, Extracellular Signal-Regulated Kinases 1 and 2; JNK, c-Jun N-terminal Kinase; P38, p38 Mitogen-Activated Protein Kinase; MAPK, Mitogen-Activated Protein Kinase; BAX, BCL-2 Associated X, Apoptosis Regulator; BCL-2, B-Cell Lymphoma 2; JaK, Janus Kinase; STAT3, Signal Transducer and Activator of Transcription 3; SOCS1/3, Suppressor of Cytokine Signaling 1/3; p-GSK 3β, Phosphorylated Glycogen Synthase Kinase 3β; HIF-1α, Hypoxia-Inducible Factor 1α; VEGF, Vascular Endothelial Growth Factor; bFGF, Basic Fibroblast Growth Factor; SPF, Serum Platelet Factor; IGF-1, Insulin-Like Growth Factor 1; IKK, Inhibitor of kappa B Kinase; IKB, Inhibitor of kappa B; TWIST, Twist-Related Protein 1; SNAIL, Snail Family Transcriptional Repressor 1; JAK2/3, Janus Kinase 2/3; RAF, Rapidly Accelerated Fibrosarcoma; SLUG, Snail Family Transcriptional Repressor 2. Figure created with BioRender.com

AXL is a member of the TAM (TYRO3, AXL, MERTK) family of RTKs, which are composed of an extracellular domain (ECD), containing tandem repeats of immunoglobulin (Ig)- and fibronectin type 3 (FN-III)-like domains, a transmembrane domain and an intracellular domain (Fig. 1). The intracellular domain contains a catalytically competent kinase defined by a unique, conserved KWIAIES sequence [14]. All TAM receptors are single-pass transmembrane receptors that bind to extracellular ligands, more specifically growth-arrest specific protein 6 (GAS6), protein S, Tubby, Tubby-like protein 1 and Galectin-3 [15–17]. Ligand-receptor interaction triggers receptor dimerization and autophosphorylation [18–20], which serves as a molecular switch, activating downstream signaling pathways that modulate cell survival, proliferation, and immune responses [21–23].

GAS6, a vitamin K-dependent protein, serves as the high-affinity ligand for the AXL receptor [19, 24]. Its name reflects the discovery in growth-arrested cells and refers to its regulating role in cellular homeostasis [25]. The specific interaction between GAS6 and AXL, facilitated by the unique carboxy-terminal “LG” (loop and ‘G’ γ-carboxyglutamic acid) domains of GAS6 and the Ig-like domains of AXL, initiates a cascade of downstream signaling pathways such as the phosphatidylinositol 3-kinase/Protein kinase B (PI3K/AKT), mitogen-activated protein kinase/extracellular signal-regulated kinase (MAPK/ERK), and Janus kinase/signal transducer and activator of transcription (JAK/STAT) pathway. Activation of these pathways orchestrate context-specific cellular responses, ranging from cell survival and proliferation to migration and differentiation [13, 23, 26]. However, evidence points to a constitutive GAS6/AXL interaction, which by itself is insufficient to trigger the activation of these downstream effectors [27]. Additionally, GAS6-independent mechanisms of AXL activation have been reported [28]. Upon AXL overexpression, excess AXL protein may lead to homophilic binding of ECDs on adjacent cells [29], or even ligand-independent homodimerization [30], with subsequent downstream activation in either case. Other studies reported ligand-independent AXL activation in response to hydrogen peroxide via reactive oxygen species (ROS) [31]. AXL also engages in crosstalk and heterodimerization with both TAM [32, 33] and non-TAM RTKs, such as the fibroblast (FGFR), epidermal (EGFR), platelet-derived (PDGFR) and hepatocyte growth factor receptors (MET), activating downstream pathways regardless the presence of the dimerization partner’s ligand (Fig. 1) [34–37]. In this way, AXL can promote resistance to several therapies, including chemotherapy and targeted therapies, or even inhibitors of its partners [38, 39].

The GAS6/AXL axis is subject to complex feedback mechanisms that regulate the intensity and duration of signaling. Negative regulators, such as protein tyrosine phosphatases [40], control AXL phosphorylation levels and prevent excessive activation, while ubiquitin-proteasome-mediated degradation ensures the timely termination of the AXL signaling cascade [41]. In addition, AXL can be cleaved by the proteases a disintegrin and metalloproteinase 10 (ADAM10) and ADAM17 (TACE), resulting in shedding of AXL into the extracellular space. This soluble fragment retains the ability to bind GAS6 through its Ig-like domains and is therefore able to function as a decoy receptor to diminish GAS6 signaling [42]. These regulatory mechanisms contribute to the precision of cellular responses, preventing aberrant signaling and maintaining cellular homeostasis. In addition, the crosstalk between AXL and GAS6 has profound implications on various physiological processes, being a finely tuned system where the balance between receptor activation, ligand availability and the presence of other signaling molecules, can elicit diverse responses tailored to specific physiological requirements [41, 43].

### Physiological role of the GAS6/AXL signaling pathway

Under physiological conditions, AXL signaling in endothelial cells, fibroblasts, smooth muscle cells and platelets contributes to angiogenesis, vascular homeostasis, extracellular matrix (ECM) protein production, and cell survival and migration, all playing a role in in tissue repair processes.

The function of AXL in immune cells is mainly studied in the context of inflammation. Using HSC-derived natural killer (NK) cells, it has been found that agonistic anti-AXL antibody or recombinant GAS6 specifically upregulated the expression of NK cell-specific receptors (e.g., LY49A, Ly49G2, Ly49C/F/I, NKG2A/C/E) and NK cell-associated molecules (e.g., IL-2Rβ, perforin, IL-15Rα, IFN-γ); suggesting a regulatory role of AXL in NK cell development, activation and effector function [44]. Under physiological conditions, AXL on phagocytic cells, including macrophages, plays a central role in apoptotic cell clearance by binding the “eat-me” signal phosphatidylserine (PS) and triggering PS-mediated efferocytosis during inflammation, which is essential for tissue development and repair. Inflammatory stimuli such as interferon (IFN) and the toll-like receptor (TLR) 3 ligand poly(I: C), upregulate AXL expression in both murine and human macrophages, enhancing the binding of AXL to GAS6, and increasing macrophage ability to engulf and clear apoptotic cells [27, 45].

In dendritic cells (DCs), AXL expression can be upregulated by inflammatory stimuli such as lipopolysaccharide. Activation of the TLR pathway results in increased AXL expression and initiates a negative feedback loop by forming a complex with the IFN-α/β receptor (IFNAR) [43]. Signaling through AXL-IFNAR induces the expression of suppressor of cytokine signaling (SOCS) 1 and 3, which in turn inhibits proinflammatory cytokine release and promotes immunosuppression to maintain tissue homeostasis. Interestingly, Villani et al.. discovered a new subset of DCs characterized by the expression of AXL and sialic acid-binding Ig-like lectin 6 (SIGLEC-6), termed AS DCs [46]. This subset was identified using single-cell RNA sequencing and showed a unique gene expression profile distinct from conventional DCs and plasmacytoid DCs. The AS DC subset exhibits high levels of genes associated with type I IFN responses, are proficient in antigen presentation and demonstrate strong responses to viral infections. The study suggested that AS DCs might originate from a common progenitor shared with other DC types but diverge to acquire their unique features. A better understanding of these AS DCs, particularly their association with type I IFN responses and potential roles in antigen presentation, could have significant implications for immunotherapies and vaccine development, particularly in the context of viral infections and cancer immunotherapy. However, further research is needed to elucidate the precise contributions of AXL in these diverse biological contexts and to determine how therapeutic AXL targeting can be optimized to mitigate disease without compromising physiological tissue repair and immune balance.

### Oncogenic role of the GAS6/AXL signaling pathway

AXL is frequently overexpressed in a variety of cancer types, and its dysregulation and activation is associated with tumor progression, poor prognosis and decreased OS [47–51]. Some of the cancers in which AXL is frequently reported to be dysregulated include breast cancer [52, 53], non-small cell lung carcinoma (NSCLC) [54–56], colorectal cancer [57], ovarian cancer [58], pancreatic cancer [59], prostate cancer [60], melanoma [61] and AML [12, 62–64]. AXL’s contribution to cancer can be attributed to its ability to activate downstream signaling cascades that play a major role in cancer cell survival, proliferation, migration/invasion, EMT, angiogenesis, and resistance to conventional therapies, as previously summarized by others [51, 65, 66]. AXL activation can drive cancer cell survival and proliferation through modulation of different signaling pathways, including the PI3K/AKT/mammalian target of rapamycin (mTOR), JAK/STAT, nuclear factor kappa-light-chain-enhancer of activated B cells (NFκB), and rat sarcoma virus/rapidly accelerated fibrosarcoma/mitogen-activated protein kinase kinase/extracellular signal-regulated kinases (RAS/RAF/MEK/ERK) pathways [67–69]. It has been found that AXL promotes tumor cell survival by regulating NFκB nuclear translocation, decreasing the activity of pro-apoptotic proteins (BAD and caspase-3) and enhancing the expression of anti-apoptotic markers (survivin, BCL-2 and BCL-XL) (Fig. 1) [70–72].

In the context of cancer cell migration and invasion, it has been shown that GAS6-induced AXL activation promotes membrane protrusions, cell motility, actin cytoskeletal remodeling, cell spreading and regulation of lysosome peripheral distribution, mainly by PI3K/AKT, MAPK/ERK, JAK/STAT and Ras-related C3 botulinum toxin substrate 1 (RAC1) activation [73–75]. In addition, AXL-induced ERK signaling contributes to enhanced cancer cell migration/invasion by promoting the expression of matrix metalloproteinases (MMPs), which degrade the ECM, facilitating cell movement [76]. AXL activation is also intimately linked with the induction of EMT, a cellular program that endows epithelial cells with mesenchymal characteristics, promoting increased motility and invasion. Through downstream effectors such as SNAIL, SLUG, and TWIST, AXL signaling represses the expression of epithelial markers (e.g., E-cadherin) and upregulates mesenchymal markers (e.g., N-cadherin, vimentin), facilitating the transition to a more invasive phenotype [47, 77, 78].

Finally, the GAS6/AXL axis also plays a significant role in promoting angiogenesis through several mechanisms, including the induction of pro-angiogenic factors (e.g., VEGF, ANG2) and stimulation of endothelial cell function [79, 80].

Taken together, AXL’s ability to drive multiple oncogenic processes makes it an attractive therapeutic target, especially in cancers characterized by poor prognosis and drug resistance. However, understanding the context-specific roles of AXL in different cancer types, its contribution to immune evasion, and how it interacts with other signaling pathways under therapeutic pressure, awaits further investigation. This could optimize current AXL-targeted therapies, especially in combination with other treatments, while considering the broader implications for normal tissue homeostasis and potential adverse effects.

### Immunoregulatory function of AXL in cancer

Besides its direct oncogenic function, AXL expression in the tumor microenvironment (TME) contributes to the creation of an immunosuppressive niche, further supporting tumor growth and therapy resistance. For example, Holtzhausen et al.. demonstrated that AXL was significantly upregulated on immunosuppressive myeloid-derived suppressor cells (MDSCs) in BRAF^V600E^/PTEN deficient melanoma tumor-bearing mice [81]. Moreover, MDSCs from tumor-bearing AXL knock-out (KO) mice failed to induce T cell suppression and migrated poorly to tumor-draining lymph nodes. A similar effect was observed upon treatment with the pan-TAM inhibitor UNC4241, which also reduced the MDSC suppressive capacity and subsequently increased CD8^+^ T cell infiltration [81].

In addition, AXL and GAS6 are both described to drive tumor-associated macrophage polarization towards an immunosuppressive, pro-tumoral, M2-like phenotype via the AXL/PI3K/AKT/NFκB pathway [82–85]. These M2 macrophages are associated with immunosuppression and tissue remodeling, mainly through secretion of anti-inflammatory cytokines and growth factors that support tumor growth and suppress immune responses. Tumor-associated macrophages with high AXL expression produce increased levels of anti-inflammatory cytokines such as interleukin-10 (IL-10) and transforming growth factor beta (TGF-β), which inhibit the activation and proliferation of effector T cells and promote the expansion of regulatory T cells (Tregs). Increased M2 polarization and AXL expression was observed upon incubation of THP-1 macrophages with tumor cell derived conditioned medium, while AXL inhibition abrogated M2 polarization. In the CT26 murine colon carcinoma model, AXL inhibition by SLC-391 decreased tumor growth and increased the ratio of M1/M2-polarized macrophages [86]. Notably, a study by Goyette et al.., using the HER2^+^ Neu FVB/NJ (MMTV-NeuNDL2-5) and NIC (MMTV-NeuNDL2-5-IRES-Cre) mouse models of breast cancer, demonstrated reduced HER2 and HIF-1α levels in AXL KO mice, especially upon hypoxia [87]. AXL was essential for HIF-1α expression during hypoxic stress, and its inhibition disrupted the hypoxic response. This suggests that AXL influences HIF-1α expression, particularly in vivo, affecting the TME and metastasis. Moreover, AXL KO had a direct impact on the activity and composition of immune cells. More specifically, a decreased infiltration of pro-tumoral CD206^+^ macrophages, neutrophils and immunosuppressive Tregs, and an increased infiltration of cytotoxic NK cells, CD45^+^ immune cells, CD8^+^ T cells, CD4^+^ T cells, and I-A/I-E^+^ (MHC class II^+^) macrophages was found in AXL KO tumors compared to AXL^+/+^ tumors.

Besides its expression on myeloid cell types, AXL was also detected on NK cells and identified as a critical target of the E3 ubiquitin ligase CBL-B (casitas B-lineage lymphoma-b) in B16F10 melanoma and 4T1 mammary tumors [88]. Genetic ablation of CBL-B or pharmacological inhibition of AXL using LDC1267 resulted in enhanced NK cell cytotoxicity, mainly by increased degranulation and the release of cytotoxic granules such as perforin and granzyme B, which are essential for the destruction of tumor cells. AXL inhibition also led to elevated secretion of pro-inflammatory cytokines, particularly IFN-γ, which is crucial for orchestrating anti-tumor immune responses. The enhanced secretion of IFN-γ highlights the broader immune-activating potential of AXL targeting in the tumor microenvironment. By boosting both direct cytotoxic activity and inflammatory signaling, AXL inhibition amplifies the anti-tumor effectiveness of NK cells. These findings were further supported by in vivo experiments, where LDC1267 treatment significantly reduced metastatic spread in murine models of melanoma and mammary cancer, emphasizing the therapeutic potential of modulating AXL activity in cancer immunotherapy. Previous work by Terry et al.. in 2019 confirmed this by demonstrating that AXL expression is correlated with increased resistance to both NK cell- and CD8^+^ T cell-mediated cancer cell killing [89]. AXL inhibition elicited a robust immune response, characterized by increased infiltration of CD8^+^ T cells and NK cells into the TME. This resulted in a heightened state of immune activation, where NK cells exhibited enhanced cytotoxic activity against mesenchymal lung cancer cells, mainly by increased expression of activation markers and cytolytic granules, such as perforin and granzyme B.

GAS6-AXL signaling was also found to affect the Treg population. GAS6 could enhance the suppressive abilities of Tregs both in vitro and in vivo, primarily through the activation of AXL [90]. This interaction between GAS6 and AXL increased the expression of forkhead box P3 (FOXP3) and cytotoxic T-lymphocyte associated protein 4 (CTLA-4), which contribute to the suppressive function of Tregs. Using an AXL KO breast cancer model (MMTV-PyMT), Aguilera et al.. observed multiple changes in the TME composition, including an increased amount of antigen-presenting myeloid DCs (CD11c^+^, MHC-II^+^) and an elevated CD8^+^ T cell infiltration [91]. This correlated with a decreased secretion of myeloid supportive cytokines (CSF1, CSF2, and CSF3), chemoattractants (CCL3, CCL4, and CCL5) and NFkB related cytokines (IL-6, TNF-a, and IL-1α).

AXL has also been implicated in the regulation of immune checkpoint molecules, including PD-L1 (CD274, PDCD1LG1 or B7-H1), on tumor cells or immune cells. Studies have shown that AXL activation can lead to the upregulation of PD-L1 expression, which in turn can inhibit the immune response by engaging PD-1 receptors on T cells and impair their activity. While the PD-L1/PD-1 axis can sometimes predict responses to anti-PD-1 therapy, its regulation is complex and influenced by various oncogenic events that enable tumor immune evasion. Research has shown that in carcinoma cells like MDA-MB-231, HeLa, and MCF7, hyperactive MERTK and AXL signaling upregulates PD-L1 expression, especially in the presence of phosphatidylserine (PS)-presenting apoptotic cells or PS-derived vesicles, partly through the PI3K/AKT pathway [92]. Boshuizen et al.. reported high PD-L1 levels in AXL-expressing tumors in the preclinical human melanoma (BLM, SkMel-147) and lung cancer (LCLC-103 H) models [93]. In the study of Sadahiro et al.., AXL inhibition using bemcentinib in murine glioblastoma resulted in decreased PD-L1 and increased PD-L2 (CD273, PDCD1LG2 or B7-DC) expression [94]. Furthermore, PD-L1 was predominantly expressed on tumor cells (CD45^−^) and myeloid cells (CD45^+^/CD11b^+^), though the specific impact of AXL targeting on these subsets requires further investigation. These findings collectively suggest a consistent association between AXL expression and PD-L1, contributing to the suppression of antitumor immune responses.

In addition, Aguilera et al.. demonstrated that the loss of AXL in the PyMT breast cancer model not only altered the immune TME, but also increased the levels of mouse MHC class I (H-2K^b^) [91]. Cancer cells typically display tumor-specific antigens or neoantigens on MHC-I molecules, which interact with CD8^+^ T cells, promoting cytotoxic T lymphocyte activity and cell-mediated lysis through the perforin/granzyme pathway or apoptosis pathways mediated by tumor necrosis factor (TNF), FAS, and TNF-related apoptosis-inducing ligand (TRAIL). Therefore, partial or complete loss of MHC-I expression can lead to tumor immune escape. Other studies reported a correlation between high AXL expression and lower MHC-I levels in lung carcinoma cell clones, though AXL inhibition did not upregulate MHC-I [89]. Notably, it did increase the expression of genes involved in antigen processing and presentation, such as *Tap1*, *Tapbp*, and *Erap2*. Further supporting the AXL/MHC-I link, a study analyzing 94 melanoma tumors, at baseline and after progression under PD-1 inhibitor treatment, found that MHC-I downregulation was associated with PD-1 inhibitor resistance [95]. Additionally, the study found associations with SNAIL upregulation and CAF signatures. TGF-β was shown to promote the expansion of AXL^high^ tumor cells and inhibit MHC-I expression, even in the presence of IFN-γ, thereby facilitating immune evasion [95]. This aligns with previous findings in prostate [96] and lung cancer cells [97], where TGF-β was identified as a repressor of MHC-I expression. Blocking TGF-β signaling could potentially overcome this immunosuppressive barrier and enhance immune responses.

Taken together, these studies across diverse models have delineated potential mechanisms underlying AXL-mediated immunosuppression, including reduced tumor antigen presentation, attenuated pro-inflammatory cytokine cascades, disrupted immune cell infiltration and increase immune checkpoint expression. While some similarities exist in reported cytokine profiles, it’s crucial to acknowledge potential differences in the immune cell populations affected, owing to tumor model-specific contexts or disparities in experimental methodologies. In essence, GAS6/AXL signaling emerges as a driver of macrophage and MDSC infiltration, and a reduced abundance of mature DCs, NK cells, as well as CD4^+^ and CD8^+^ T lymphocytes. This comprehensive understanding underscores the pivotal role of AXL in shaping immune responses and highlights its potential as a therapeutic target in immunomodulatory strategies against cancer and other immune-related disorders. However, the exact mechanisms by which AXL modulates immune checkpoints and how it can be best targeted in combination with immunotherapies is yet to be determined. Addressing this could unlock new therapeutic avenues to enhance immune responses and overcome resistance in cancer treatment.

## AXL in Acute myeloid leukemia

### AXL expression and its prognostic value in AML

In 1991, O’Bryan et al.. reported the finding of an unexplained transforming gene in two chronic myeloid leukemia (CML) patients, marking the first discovery of AXL for the diagnosis of a chronic myeloproliferative disease (CMPD) [14, 39]. Three years later, AXL was linked for the first time to AML, when a study by Neubauer et al.. showed that AXL could be detected primarily in cells derived from myeloid versus lymphoid malignancies [98]. More specifically, AXL mRNA levels were observed in hematopoietic CD34^+^ progenitor and BM stromal cells (BMSC), and to a lesser extent in peripheral blood monocytes in 59% of patients with myeloproliferative disorders (39/66 cases). In 1999, a multicenter trial of the Swiss group for Clinical Cancer Research revealed that AXL expression in AML was associated with adverse prognosis but is not confined to a single subclass of the French-American-British (FAB) classification [62]. AXL expression was reported in 35% of the AML patients (19/54 cases) and was associated with a worse progression-free survival (PFS) and OS. Interestingly, a correlation was found between AXL and BCL-2 expression levels, and AXL transcript numbers were also higher in AML patients with high CD34 expression. No other correlations based on patient’s age, FAB category, or presence of extramedullary disease were found.

Using 112 samples of cytogenetically normal AML patients, Ben-Betalla et al.. observed AXL and GAS6 expression in 57% and 90% of the patients, respectively [12]. Moreover, it was found that patients expressing AXL above the median correlated with a worse OS, while GAS6 lacked prognostic value. The latter was refuted in a study by Whitman et al.. which investigated the prognostic relevance of GAS6 expression in 270 adults with de novo cytogenetically normal AML [11]. They were the first to report that GAS6 expression in patients, especially those aged ≥ 60 years, predicted failure to achieve CR and was correlated with a shorter disease-free survival and OS. Additionally, Yang et al.. reinforced the prognostic significance of GAS6 by demonstrating that higher GAS6 expression correlated with shorter event-free survival and OS in AML patients undergoing allogeneic HSC transplantation [99]. Consistently, the study of Tirado-Gonzalez et al.. revealed that high GAS6 expression correlated with poor AML patient outcomes and that patient-derived leukemic cells enhanced GAS6 expression in CD14^+^ monocytes from healthy donors [100]. Collectively, these studies underscore the significance of GAS6/AXL signaling in AML progression and its potential as a prognostic biomarker. The correlation between AXL and BCL-2, as well as the enrichment of AXL in CD34^+^ progenitors, points to its involvement in maintaining leukemic stem cell (LSC) populations, which may contribute to disease persistence and relapse. Understanding the precise mechanisms by which AXL and GAS6 promote leukemogenesis and resistance, especially in the context of genetic and molecular heterogeneity in AML needs clarification. Moreover, monitoring AXL expression in cancer patients will allow oncologists to identify which patient subgroups would benefit most from AXL-targeted therapies and could pave the way for a more personalized and effective treatment strategy for, among others, AML patients.

### AXL signaling in leukemic stem cells and AML cells in the BM niche

Although GAS6 is only expressed at low levels in AML cells, Ben-Betalla et al.. identified a paracrine interaction between AML cells and GAS6-expressing BMSC. In this paracrine loop, AML cells induce GAS6 expression in BMSC, which in turn increases AXL activation and tumor cell survival [12]. Using bemcentinib, it has been found that blocking GAS6/AXL interaction attenuated AKT and MAPK signaling in MV4-11 AML cells in vitro. Furthermore, increased protein levels of the pro-apoptotic p53 upregulated modulator of apoptosis (PUMA) and decreased protein levels of BCL-2, p-AKT and p-ERK levels were observed upon bemcentinib treatment of AML cells [12]. In an indirect way, knock-down of ALKBH5, a N^6^-methyladenosine (m6a) demethylase, was found to downregulate AXL expression and reduced the phosphorylation of downstream signaling pathways, including p-SRC, p-AKT, p-ERK1/2, p-STAT3, and p-PLCγ (phospholipase C gamma), which were rescued upon restoration of ALKBH5 expression [101].

Besides its oncogenic activity in AML cells, AXL was found to contribute to AML progression and relapse by supporting the maintenance and survival of LSCs through the lysine demethylase 4 C (KDM4C)-ALKBH5-AXL signaling axis [101]. LSCs are a subset of leukemic cells characterized by their self-renewal capacity and typical resistance to conventional chemotherapies. KDM4C expression in LSCs was found to increase chromatin accessibility by reducing H3K9me3 and recruiting MYB and Pol II to the ALKBH5 promoter. ALKBH5 subsequently promoted the expression of AXL and further activated downstream signaling pathways including PI3K, MAPK, JAK/STAT, and NFκB, this way contributing to LSCs survival and AML progression. Furthermore, the study of Niu et al.. revealed significantly elevated AXL levels in CD34^+^ stem/progenitor cells from AML patients at diagnosis [64]. Interestingly, higher AXL/GAS6 levels could be detected in stem cell-enriched Lin^–^CD34^+^CD38– cells from AML patients harboring mixed lineage leukemia (MLL) fusions compared to non-MLL samples. While all these data implicate a function of AXL in LSC survival, the impact of AXL-targeting compounds on this specific cell population, which are crucial for long-term disease control, remains unclear. Additionally, understanding the broader effects of AXL-targeting strategies within the leukemic microenvironment could contribute to more effective therapies that address both tumor and microenvironmental survival signals.

### AXL as a regulator of AML cell drug resistance

The development of drug resistance in leukemia cells constitutes the major reason for treatment failure in AML patients. In solid tumors as well as in hematological malignancies, AXL expression has been linked with increased resistance to chemotherapy, immunotherapy and targeted therapies [102–107]. In 2008, Hong et al.. compared the protein tyrosine kinase expression in drug-sensitive, before the administration of chemotherapy, and drug-refractory samples from the same AML patient [72]. AXL was found to be consistently overexpressed in the drug-resistant AML samples compared to the drug-sensitive AML samples. The effect of the chemotherapeutics doxorubicin, VP16 and cisplatin on AXL expression was studied using U937 AML cells and demonstrated chemotherapy-induced AXL upregulation and phosphorylation in vitro. Moreover, it was found that both GAS6 and AXL were required to induce chemotherapy resistance and was associated with increased expression of BCL-2 and TWIST. These findings were further supported by Ben-Betalla et al.., who demonstrated that the chemotherapeutics cytarabine and doxorubicin could upregulate AXL expression in GAS6^+^ AML cell lines MV4-11 and OCI-AML5, while this effect was absent for the GAS6– cell line HL60. Combining chemotherapeutics with bemcentinib significantly reduced the growth of MV4-11 cells in vitro and in vivo, while no additive effects could be observed using AKT or MAPK inhibitors in combination with bemcentinib. More recently, our group also demonstrated that Fc-conjugated AXL-targeting single domain antibodies (sdAbs) acted synergistically in combination with the standard-of-care agent cytarabine in THP-1 and MOLM-13 AML cell lines [108]. All these data underscore the AXL-mediated impact on chemoresistance and the potential of combining AXL inhibitors and chemotherapeutics in AML patients.

In addition to its role in chemoresistance, AXL overexpression also mediates resistance to various targeted therapies, including FLT3-targeted therapies [109]. In 2009, a study by Park et al.. reported that AXL is crucial for optimal signaling and biological activities of c-Kit in human CD34^+^ hematopoietic progenitor cells [110]. As c-Kit is a RTK from the same type III RTK family as FLT3, these data suggested that AXL could play a role in the regulation of FLT3 signaling. Further studies demonstrated that AXL activation contributed to a constitutive FLT3-ITD phosphorylation and activation [111]. FLT3 inhibitors midostaurin (PKC412) and quizartinib (AC220) enhanced AXL, ERK and AKT phosphorylation, and also STAT5 in the case of midostaurin. Using primary blasts of FLT3-ITD^+^ AML patients, it was found that midostaurin-sensitive cells harbored little phosphorylated AXL, while most midostaurin-resistant AML cells possessed a significantly higher level of AXL phosphorylation. Treatment of midostaurin-resistant MOLM-13 (FLT3-ITD^+^) AML cells with the AXL inhibitor TP-0903 or soluble AXL chimeric protein Axl-Fc restored sensitivity to both midostaurin and quizartinib, suggesting an important role of AXL in the resistance of FLT3-ITD^+^ AML cells against the FLT3 inhibitors. Notably, Axl-Fc was also able to inhibit cell growth and induce cell-cycle arrest and apoptosis in MV4-11 and MOLM-13 AML cells. A study by Dumas et al.. provided evidence that stromal cell-secreted GAS6 activated AXL and contributed to AML progression and resistance to the FLT3-inhibitor quizartinib [112].

Although AXL expression is not directly associated with resistance to BCL-2 inhibitors, a study by Niu et al.. revealed a strong synergistic effect of the BCL-2 inhibitor venetoclax and the AXL inhibitor SLC-391 in primary AML patient samples and patient-derived xenograft models [64]. These effects were mainly attributed to a significant downregulation of oxidative metabolism. Additive effects were also observed by our group using the combination of venetoclax and Fc-conjugated AXL-targeting sdAb20 in THP-1 and MOLM-13 cells [108]. The combination of AXL inhibitor ONO-7475 and venetoclax demonstrated significant synergistic effects in FLT3-ITD^+^ MV4-11 and MOLM-13 AML cell lines, enhancing apoptosis and reducing cell viability compared to single agent therapy [113]. Using primary FLT3-ITD AML samples, Post et al.. showed that ONO-7475 as a monotherapy showed limited efficacy, and venetoclax alone had minimal impact on apoptosis in certain primary samples, whereas their combination synergized to activate apoptosis. This underscores the importance of combinatorial approaches to effectively target AML with heterogeneous responses.

### AXL-mediated immunosuppression in AML

Studies demonstrating that AXL inhibitors restore drug sensitivity, especially in combination therapies, suggest that targeting AXL could mitigate resistance and improve treatment outcomes. However, the precise mechanisms through which AXL influences various resistance pathways, particularly within the bone marrow microenvironment, and its broader effects on AML cell metabolism and immune evasion remain areas requiring further investigation. In their comprehensive study, Tirado-Gonzalez et al.. conducted an in-depth investigation into the effects of GAS6/AXL pathway inhibition on leukemia cell growth and the immune response, using the ASXL1 and MLL-ENL AML mouse models [82]. The researchers employed a GAS6-deficient immunocompetent mouse model (GAS6^−/−^) and observed a substantial reduction in leukemic burden and significantly prolonged survival rates upon GAS6 absence. These beneficial effects were not observed in immunocompromised mice, underscoring the critical role of the immune system in mediating these effects. To dissect the contributions of different immune cell types, the study employed conditional KO models using NSG mice. While selective AXL deletion in DCs, using a CD11c-eGFP-Cre line, did not result in increased anti-leukemic immunity, depletion of AXL in macrophages using Csf1r-Cre^+^ Axl^f/f^ mice did increase anti-leukemic effects and prolonged survival of AML models. This macrophage-specific AXL deletion not only prolonged survival in AML models, but also resulted in heightened activation of both T cells and NK cells, as characterized by increased production of pro-inflammatory cytokines (IL-12, TNF-α), effectively targeting and eradicating both naïve and treatment-resistant leukemia cells.

Using an anti-NK1.1 antibody, the researchers reported similar leukemic burdens in both NK-depleted and non-depleted Axl^f/f^ control animals, indicating functional impairment of NK cells. However, NK cell depletion in Csf1r-Cre^+^ Axl^f/f^ mice abolished anti-leukemic immunity and led to faster disease progression, demonstrating that AXL inhibition in phagocytes triggers a robust NK cell response that is crucial for leukemia clearance. By generating mice that lacked both CD8^+^ T cells and AXL expression in phagocytes (Csf1r^−^Cre^+^ Axl^f/f^ CD8a^−/−^ and Axl^f/f^ CD8a^−/−^), it was showed that the survival benefit conferred by AXL-deficient phagocytes persisted even in the absence of CD8^+^ T cells. This indicates that other immune cells play a significant role in mediating the effects of AXL inhibition.

To further assess the therapeutic potential of AXL inhibition, leukemia-bearing mice were treated with bemcentinib on an intermittent schedule (5 days on, 2 days off). Bemcentinib significantly increased OS in the Asxl1^−/−^ model, but lost this effect in immunocompromised NSG mice, which is in line with the drug’s expected impact on AXL-positive immune cells.

## AXL-targeted therapies in preclinical and clinical evaluation for AML

The recognition of AXL as a critical mediator of AML pathogenesis has spurred the development of AXL-targeted therapies aimed at disrupting AXL signaling and sensitizing leukemia cells to conventional chemotherapy. Small molecule selective inhibitors (Table 1), multitargeted inhibitors (Tables 1 and 2), antibody-drug conjugates (ADC) (Table 3) and anti-AXL-Fc fusion proteins (Table 3) have shown promising preclinical efficacy towards AML, attenuating leukemic cell proliferation, inducing apoptosis, and enhancing the efficacy of standard chemo- and immunotherapeutic agents. Additionally, combinatorial approaches using AXL inhibitors in conjunction with targeted agents against other dysregulated pathways in AML, such as FLT3 inhibitors or BCL-2 inhibitors, hold potential for overcoming therapeutic resistance.

**Table 1:** Overview of type I AXL inhibitors and their current status in clinical trials for AML

Drug	Developer	Target(s)^a^	IC_50_ for AXL	Clinical Trial No.^b^	Phase of approval	Indications	Monotherapy/ combinations	Status
BGB324 (R428/ Bemcentinib)	Rigel Pharmaceuticals/BerGen BIO	AXL (selective)	14 nM (in vitro) 14 nM (in cells)	NCT02488408	Ib/II	AML, MDS	± Cytarabine/ Decitabine	Unknown
TP-0903 (Dubermatinib)	Tolero Pharmaceuticals	AXL, FLT3	27 nM (in vitro)	NCT03013998	Ib/II	AML	Biomarker-based multidrug therapy	Recruiting
Gilteritinib (ASP2215, Xospata^®^)	Astellas Pharma/ Kotobuki Pharmaceutical	FLT3, AXL	0.73 nM (in vitro)	NCT02421939	III	R/R FLT3-mutated AML	Gilteritinib vs. LoDAC, FLAG-IDA, MEC & Azacitidine	Active, not recruiting
				NCT02752035	III	De novo FLT3-mutated AML	± Azacitidine	Active, not recruiting
				NCT03182244	III	R/R FLT3-mutated AML	Gilteritinib vs. LoDAC, MEC & FLAG	Active, not recruiting
				NCT04293562	III	De novo FLT3-mutated AML	Multidrug treatment	Active, not recruiting
				NCT05177731	III	AML	Multidrug treatment	Active, not recruiting
				NCT02115295	II	AML, high-risk MDS or Blastic Phase CML	Multidrug treatment	Recruiting
				NCT05955261	II	Pedriatric AML	Multidrug treatment	Recruiting
				NCT06022003	II	R/R FLT3-mutated AML	+ Azacitidine	Recruiting
				NCT06221683	II	AML	Multidrug treatment	Recruiting
				NCT06317649	II	De novo FLT3-mutated AML	+ Venetoclax & Azacitidine	Not yet recruiting
				NCT03013998	Ib/II	AML	Biomarker-based multidrug	Recruiting
				NCT05279859	Ib/II	R/R FLT3-mutated AML	+ ERAS-007/ ERAS-601	Withdrawn
				NCT05028751	Ib/II	R/R FLT3-mutated AML	+ Lanraplenib	Terminated
				NCT02310321	I/II	De novo AML	+ Cytarabine/ Idarubicin	Active, not recruiting
				NCT04140487	I/II	FLT3-mutated AML or MDS	+ Venetoclax & Azacitidine	Active, not recruiting
				NCT04240002	I/II	R/R FLT3-mutated AML	+ FLAG	Recruiting
				NCT05010122	I/II	R/R FLT3-mutated AML or high-risk MDS	+ Venetoclax/ Decitabine & Cedazuridine	Recruiting
				NCT05520567	I/II	De novo FLT3-mutated AML	+ Venetoclax/ Azacitidine	Recruiting
				NCT06235801	I/II	R/R FLT3-mutated AML	+ Momelotinib	Recruiting
				NCT04336982	Ib	De novo or R/R AML	+ CC-90,009/ Venetoclax/ Azacitidine	Terminated
				NCT05010772	Ib	AML in remission	+ Decitabine & Cedazuridine	Recruiting
				NCT04655391	Ib	AML Post AHCT	+ Glasdegib	Withdrawn
				NCT05330377	Ib	R/R FLT3-mutated AML	+ Mitoxantrone/ Cladribine/ Cytarabine/ Filgrastim	Withdrawn
				NCT05756777	Ib	R/R FLT3/IDH1 or FLT3/IDH2-mutated AML	+ Ivosidenib/ Enasidenib	Recruiting
				NCT05024552	I	R/R FLT3-mutated AML	+ Vyxeos	Recruiting
				NCT05546580	I	R/R FLT3-mutated AML	+ Iadademstat	Recruiting
				NCT06222580	I	R/R FLT3-mutated AML with MLL or NPM1 mutation	+ Revumenib	Recruiting
				NCT06001788	I	R/R AML with KMT21- or NPM1-mutation	+ Ziftomenib	Recruiting
				NCT05312112	Observational	AML	± Venetoclax	Recruiting
				NCT04691648	Observational	R/R FLT3-mutated AML	Monotherapy	Recruiting
				NCT06265545	Platform trial	R/R FLT3-mutated AML	+ Ivosidenib/ Venetoclax/ Selinexor	Not yet recruiting

*Abbreviations MDS*: Myelodysplastic syndromes, *R/R*: Relapsed or refractory, *FLT3*: Fms-like tyrosine kinase 3, *AHCT*: Autologous hematopoietic cell transplantation, *ITD*: Internal tandem duplication, *CML*: Chronic myeloid leukemia, *IDH1/2*: Isocitrate dehydrogenase 1/2, *NPM1*: Nucleophosmin 1, *ALL*: Acute lymphoblastic leukemia

^a^In the order of inhibition potency

^b^All the relevant information of clinical trials can be found on the public clinical trial registry website (clinicaltrials.gov)

**Table 2:** Overview of type II AXL inhibitors and their current status in clinical trials for AML

Drug	Developer	Target(s)^a^	IC_50_ for AXL	Status
Cabozantinib (XL184, Cabometyx, BMS-907351, Cometriq^®^)	Exelixis/Ipsen company	VEGFR2, MET, RET, KIT, AXL, FLT1/3/4	7 nM (in vitro), 42 nM (in cells)	Terminated phase Ib trial^b^ (NCT03878524)
Foretinib (XL880, EXEL-2880, GSK1363089)	GSK	MET, VEGFR2, TIE-2, VEGFR3, RON, AXL	11 nM (in vitro), < 100 nM (in cells)	Preclinical
Sitravatinib (MGCD516)	Mirati Therapeutics Inc.	DDR2, EPHA3, AXL, MER, VEGFR3	1.5 nM (in vitro), 250–800 nM (in cells)	Preclinical
Rebastinib (DCC-2036)	Deciphera Pharmaceuticals LLC	ABL, FLT3, VEGFR2, TIE-2, Lyn, SRC, FGR, AXL	42 nM (in vitro)	Preclinical

^a^In the order of inhibition potency

^b^All the relevant information of clinical trials can be found on the public clinical trial registry website (clinicaltrials.gov)

**Table 3:** Other AXL inhibitors, anti-AXL monoclonal antibodies and soluble receptors in preclinical development for AML

Drug	Drug class	Target(s)^a^	Clinical stage
UNC569	Small molecule inhibitor	MERTK, AXL	Preclinical
CTS2016	Small molecule inhibitor	AXL, FLT3	Preclinical
SLC-391	Small molecule inhibitor	AXL, TYRO3, MERTK	Preclinical
DAXL-88	Antibody	AXL, FLT3	Preclinical
DAXL-88-MMAE	Antibody-drug conjugate	AXL, FLT3	Preclinical
sdAb20-Fc	Single domain antibody	AXL	Preclinical
Axl-Fc	Soluble receptor	GAS6	Preclinical
MYD1-72	Decoy receptor	GAS6	Preclinical

^a^In the order of inhibition potency

### Small molecule inhibitors

**Fig. 2:**
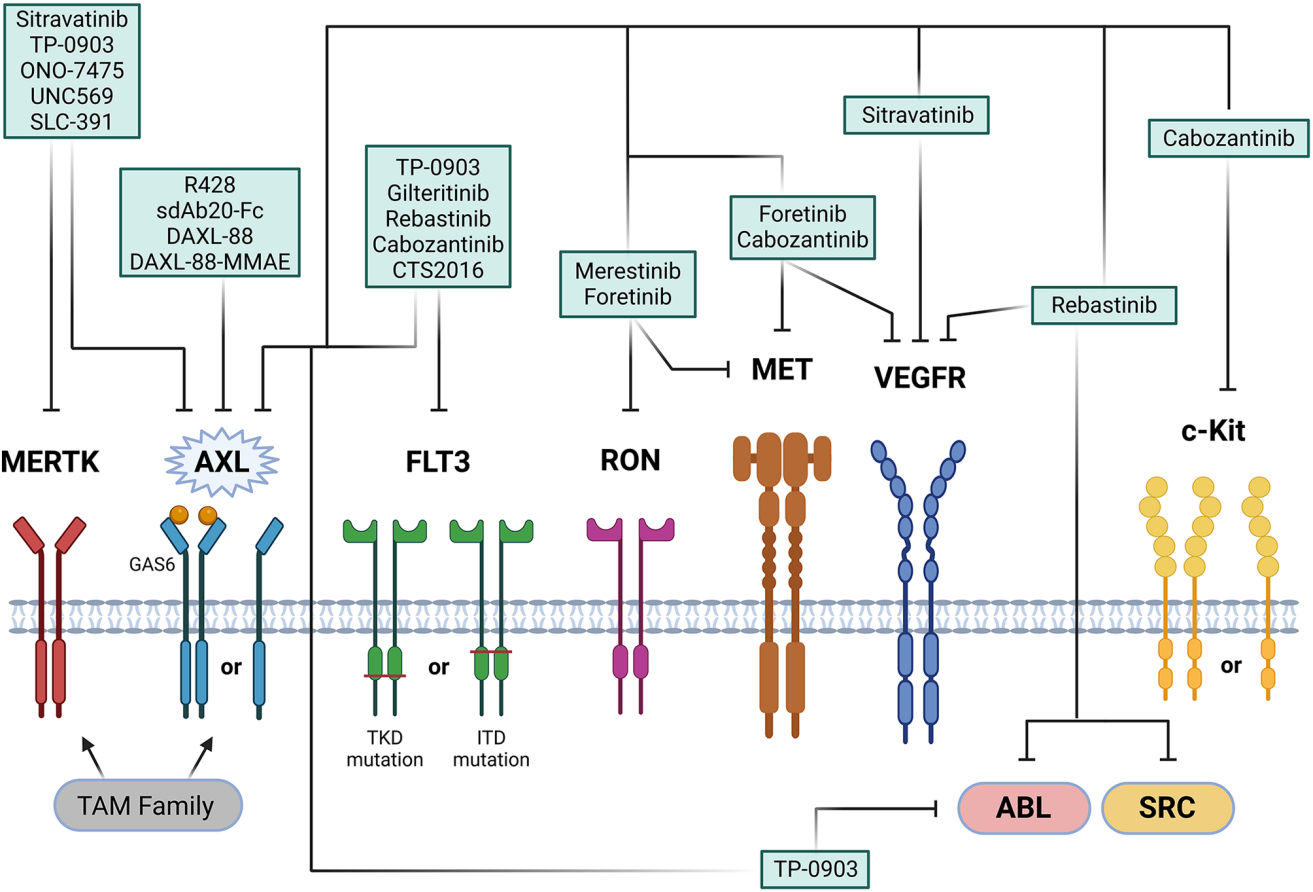
AXL-targeted therapies in (pre)clinical evaluation for AML Summarizing figure of all AXL-targeted therapies in AML, including small molecule inhibitors, antibody-drug conjugates (ADCs), anti-AXL-Fc fusion proteins, and inhibitors targeting multiple receptors and/or proteins. These agents have demonstrated significant (pre)clinical efficacy by reducing leukemic cell proliferation and inducing apoptosis, thereby boosting the effectiveness of standard chemo- and immunotherapy. FLT3, FMS-like Tyrosine Kinase 3; RON (MST1R), Macrophage Stimulating 1 Receptor; MET, Mesenchymal to Epithelial Transition factor (also known as Hepatocyte Growth Factor Receptor, HGFR); VEGFR, Vascular Endothelial Growth Factor Receptor; c-KIT, v-kit Hardy-Zuckerman 4 Feline Sarcoma Viral Oncogene Homolog (also known as CD117); ABL, Abelson Murine Leukemia Viral Oncogene Homolog 1; SRC, Proto-Oncogene Tyrosine-Protein Kinase; TKD, Tyrosine Kinase Domain; ITD, Internal Tandem Duplication. Figure created with BioRender.com

The past years, various small molecule inhibitors targeting AXL have been evaluated for the treatment of AML (Tables 1, 2 and 3; Fig. 2). Interestingly, most of these inhibitors are multikinase inhibitors and often exhibit broad-spectrum activity, targeting kinases such as TAM family members TYRO3 and MERTK; as well as other RTKs such as vascular endothelial growth factor receptor (VEGFR), MET, FLT3, recepteur d’origine nantais (RON), and AURORA A/B in addition to AXL, which can contribute to their therapeutic efficacy in AML. AXL inhibitors can be classified into two main categories, namely type I and type II inhibitors. Type I inhibitors bind to the active “aspartate-phenylalanine-glycine (DFG)-in” conformation of the kinase, interacting with the ATP-binding site when the kinase is in its “on” state. Conversely, type II inhibitors prefer the inactive DFG-out conformation of the kinase, targeting an adjacent allosteric site that is exposed only when the kinase is “off” [114, 115]. This distinction is crucial as it influences the binding affinity, selectivity, and overall therapeutic potential of the inhibitors. Several AXL inhibitors have shown promise in preclinical studies and have advanced into different stages of clinical investigation [116–118]. These inhibitors, by targeting multiple pathways and conformations, hold potential for enhancing therapeutic outcomes and overcoming resistance mechanisms in AML treatment.

## Type I AXL inhibitors

### Bemcentinib (BGB324, R428; Rigel Pharmaceuticals/BerGenBio)

Bemcentinib is a first-in-class, oral, specific and highly selective AXL kinase inhibitor and is by far the most advanced anti-AXL therapeutic agent [119]. The activity of bemcentinib is limited to the tyrosine kinase subfamily, with the highest inhibitory potency for AXL among all 133 kinases [120]. It has been reported that bemcentinib blocks the catalytic activities of AXL, at nanomolar concentrations (IC_50_ = 14 nM), reduces AXL and p-AXL levels, induces the accumulation of autophagosomes and lysosomes, blocks lysosomal acidification and recycling, and increases apoptosis of tumor cells [121]. In addition, Ben-Betalla et al.. reported that, when used alone or in combination with cytotoxic agents, bemcentinib abrogated the AKT and MAPK pathways by upregulating PUMA and suppressing BCL-2 [12]. Bemcentinib was the first AXL-specific inhibitor to enter clinical investigation and is currently in phase I/II clinical trials, either alone or in combination with other therapies, for the treatment of AML, myelodysplastic syndromes (MDS), triple-negative breast cancer (TNBC), glioblastoma, NSCLC, metastatic melanoma and malignant mesothelioma (NCT03965494, NCT02922777, NCT02872259, NCT03184558, NCT03184571, NCT02488408, NCT02424617, NCT03824080 and NCT03654833). Loges et al.. reported that bemcentinib exhibited the potential for safe administration over prolonged periods, effectively suppressing AXL signaling and demonstrating anti-leukemic activity [122]. Bemcentinib was well tolerated as a monotherapy and in combination with low-dose cytarabine (LDAC) or decitabine in the phase Ib/II study BGBC003 (NCT02488408), after which bemcentinib was given FDA fast-track designation for the treatment of elderly patients with relapsed/refractory (R/R) AML [123, 124]. Further evaluation in the BERGAMO phase II trial (NCT03824080) showed bemcentinib’s moderate efficacy in high-risk R/R MDS/AML, with a 24% overall response rate, and a higher response in MDS patients (44%) compared to AML patients (11%) [125]. Although exploratory analysis suggested that patients with STAG2 mutations may respond better to bemcentinib, further research is needed to refine patient selection and improve outcomes, potentially by combining bemcentinib with other therapies.

### Dubermatinib (TP-0903; Tolero Pharmaceuticals)

TP-0903 is a potent AXL inhibitor with high-affinity, oral bioavailability and concurrent FLT3 inhibitory activity. With an in vitro IC_50_ of 27 nM [126, 127], TP-0903 disrupts AXL phosphorylation, reverses EMT, and increases the depletion of anti-apoptotic proteins MCL-1, X-linked inhibitor of apoptosis protein (XIAP), and BCL-2, fostering dose-dependent apoptosis of primary chronic lymphocytic leukemia (CLL) B-cells, even in cases with adverse prognostic factors like 17p/p53 deletions or prior exposure to agents like ibrutinib [128, 129]. TP-0903 also demonstrated promising efficacy against *de novo* and drug-resistant FLT3-ITD^+^ AML, both in vitro and in vivo [130]. Furthermore, Park et al.. reported that TP-0903 resensitized MOLM-13 FLT3-ITD^+^ AML cells to the FLT3-inhibitors midostaurin and quizartinib [111]. However, as TP-0903 was found to also target aurora kinase and JAK2, it could not be ruled out that abrogation of FLT3-ITD^+^ AML resistance was due to off-target activities of TP-0903. Jeon et al.. observed a correlation between TP-0903’s potency and STAT, AKT, and ERK signaling, alongside cell cycle regulators in both biochemical and cellular assays [131]. Their findings demonstrated that TP-0903 exhibited ex vivo activity in primary AML cells characterized by recurrent mutations like MLL-PTD, ASXL1, SRSF2, and WT1, which are known to be associated with unfavorable prognosis or to contribute to clinical resistance against AML-directed therapies. In TP53 mutant AML cell lines, TP-0903 effectively inhibited cell viability (IC_50_ = 12 − 32 nM), induced apoptosis at 50 nM, and upregulated pChk1/2 and pH2AX, indicating DNA damage induction [132]. Moreover, the combination of TP-0903 and decitabine showed additive effects in vitro and significantly prolonged median survival in mouse models xenografted with TP53 mutant AML compared to single-agent treatments. Clinical trials regarding TP-0903 as a monotherapy include patients with CLL, refractory solid tumors and FLT3 mutated AML (NCT03572634, NCT02729298 and NCT04518345). In addition, the ongoing Beat AML phase 1b/2 study of TP-0903 and decitabine targeting mutant TP53 and/or complex karyotype in patients with untreated AML (NCT03013998) reported a composite CR (CRh/Cri) rate of 66% achieving minimal residual disease negativity at dose level 1, leading to selection of this dose as the recommended phase 2 dose [118].

### Gilteritinib (ASP2215; Astellas Pharma/Kotobuki Pharmaceutical)

Gilteritinib is a highly specific inhibitor of FLT3, AXL and anaplastic lymphoma kinase (ALK or CD246), exhibiting significant anti-leukemic activity in patients with R/R AML. Targeting both AXL and FLT3 with gilteritinib results in tumor regression and decreased proliferation in FLT3 mutation-positive AML models, both in vitro and in vivo [133]. Dumas et al.. reported that gilteritinib possessed anti-proliferative and pro-apoptotic effects on FLT3-ITD^+^ MOLM-13, MOLM-14 and MV4-11 AML cell lines [134]. These effects were associated with reduced phosphorylation of FLT3 and AXL, as well as decreased activation of downstream signaling pathways. The study indicated that dual FLT3/AXL inhibition by gilteritinib provided more effective inhibition of FLT3-ITD^+^ cells, compared to the highly selective FLT3 inhibitor quizartinib, under conditions mimicking the BM microenvironment, such as hypoxia and co-culture with BM stromal cells. However, the enhanced efficacy of gilteritinib may partly be attributed to differences in dosing, and additional studies are needed to confirm whether AXL inhibition specifically contributes to this effect. Notably, the study observed that AXL expression and its ligand GAS6 are upregulated in hypoxic conditions and in BM stromal cell co-culture.

Early phase I/II trials demonstrated the anti-leukemic efficacy and tolerable side effects of gilteritinib in R/R AML patients across the USA, Germany, Italy, and Japan (NCT02181660 and NCT02014558) [116]. The multicenter, randomized phase III ADMIRAL trial (NCT02421939) confirmed that gilteritinib significantly improves OS and CR rates compared to chemotherapy, with fewer adverse events and a comparable safety profile, in R/R AML patients with FLT3 mutations, leading its FDA approval for this indication [135–137]. Current research includes exploring gilteritinib in combination with other therapies, such as venetoclax (NCT03625505), which has demonstrated high composite CR rates, and atezolizumab (NCT03730012), which, despite an acceptable safety profile at lower doses, exhibited dose-limiting toxicities at higher doses [138, 139]. However, these findings support continued investigation into gilteritinib-based combination therapies. Therefore, gilteritinib is currently being evaluated in several clinical trials on AML patients, including in combination with gemtuzumab ozogamicin (Mylotarg^®^; NCT06022003), azacytidine (NCT06022003, NCT04140487, NCT02752035), atezolizumab (NCT03730012), ivosibenib/enasidenib (NCT05756777), iadademstat (NCT05546580), decitabine (NCT05010772), momelotinib (NCT06235801), revumenib (NCT06222580), CC-90,009 (NCT04336982), venetoclax/azacytidine (NCT05520567, NCT04140487), cytarabine/daunorubicin (Vyxeos^®^; NCT05024552) and venetoclax/decitabine (NCT05010122).

### Tamnorzatinib (ONO-7475; Ono Pharmaceutical Co., Ltd.)

The dual AXL/MERTK inhibitor ONO-7475 has demonstrated efficacy in targeting FLT3-mutant AML cells by inhibiting key signaling pathways involving ERK and MCL-1, which are implicated in drug resistance against venetoclax [113]. Combination treatment of ONO-7475 with venetoclax exhibited potent cytotoxicity against FLT3-mutant AML cell lines and primary cells, including those resistant to venetoclax, particularly those overexpressing MCL-1 [140]. In vivo studies demonstrated that ONO-7475 monotherapy significantly prolonged survival in AML cell lines and PDX models [113]. In short, the combination of ONO-7475 and venetoclax surpassed the efficacy of ONO-7475 monotherapy, significantly reducing leukemic burden and extending survival in both model systems. Based on these findings, ONO-7475 entered clinical evaluation to assess its safety and tolerability as monotherapy in patients with R/R AML or MDS and to investigate its efficacy in combination with venetoclax (NCT03176277). However, this study was terminated in May 2024 due to its ineffectiveness.

## Type II AXL inhibitors

### Cabozantinib (XL184; Exelixis/Ipsen company)

Cabozantinib is a non-selective multikinase inhibitor that targets multiple RTKs, including VEGFR2, c-MET, KIT (also known as CD117), RET, AXL, ROS1 and FLT3, and the related angiogenesis and metastasis processes. It has been demonstrated that cabozantinib induces apoptosis in FLT3-ITD^+^ leukemia cells in a dose-dependent manner [141]. However, FLT3-ITD lacking leukemia cell lines were resistant to cabozantinib. A clinical trial (NCT01961765) revealed favorable tolerability of cabozantinib in AML patients, with notable potency observed as an inhibitor of FLT3/ITD-altered tyrosine kinases [142]. Other phase III trials are now ongoing in, among others, differentiated thyroid cancer (NCT03690388), meningioma (NCT05425004), recurrent liver cancer post-transplant (NCT04204850), locally advanced kidney cancer (NCT04022343) and carcinoid tumors (NCT03375320).

### Merestinib (LY2801653; Eli Lilly and Company/Dana-Farber Cancer Institute)

Merestinib (LY2801653) is a dual MET/AXL inhibitor that also targets RON, FLT3, MERTK, angiopoietin-1 receptor (also known as CD202B or TEK), ROS1, discoidin domain receptor family member 1/2 (DDR1/2), and the MAP kinase-interacting serine/threonine-protein kinases 1/2 (MKNK1/2) [143]. Merestinib potently blocks AXL and MET phosphorylation, and the subsequent activation of their downstream signaling molecules, and has demonstrated anti-leukemic effects by effective blockade of eukaryotic translation initiation factor 4E (eIF4E) phosphorylation in AML cells [144]. Additionally, Kosciuczuk et al.. demonstrated the suppression of early leukemic progenitors derived from AML patients both in vitro and in a xenograft mouse model of AML. A single-center, nonrandomized, open-label phase I clinical trial in R/R AML patients (NCT03125239) has been completed and reported reversible grade 3 transaminase elevation as the primary dose-limiting toxicity of merestinib, either alone or in combination with LY2874455 [145]. Despite this, several patients experienced stable disease, with one achieving CR without measurable residual disease. Single agent merestinib showed safety and biological activity, with correlative studies indicating therapeutic plasma levels, effective attenuation of MET signaling, and increased hepatocyte growth factor expression in BM aspirate samples of refractory leukemia patients.

### Foretinib (XL880, EXEL-2880, GSK1363089; GSK)

Foretinib is an oral multikinase inhibitor that targets several key kinases, including AXL, MET, VEGFR, ROS, RON, and TIE-2 [146]. By blocking AXL phosphorylation, foretinib effectively suppresses cell proliferation, dissemination, and survival, leading to inhibition of in vivo tumor growth [57]. In the context of AML, Wang et al.. reported that foretinib exhibited superior efficacy compared to existing FLT3 inhibitors in patients with FLT3-ITD mutations [147]. Foretinib directly bound to FLT3, effectively inhibiting its signaling pathway, resulting in potent anti-proliferative and pro-apoptotic effects in AML cell lines and primary AML cells harboring FLT3-ITD mutations. Notably, foretinib also showed activity against secondary FLT3-ITD mutations resistant to quizartinib and gilteritinib. Several clinical trials for the treatment of recurrent/metastatic breast cancer, HCC, NSCLC, metastatic gastric cancer, papillary RCC and squamous cell cancer, have been conducted or are still ongoing and have reported antitumor activity of foretinib, with partial responses and stable disease [148].

### Sitravatinib (MGCD516; Mirati Therapeutics Inc.)

Sitravatinib inhibits a closely related group of RTKs, including KIT, PDGFRα, PDGFRβ, AXL and MET [149]. Zhang et al.. demonstrated that sitravatinib reduced cell proliferation, induced cell cycle arrest, and increased apoptosis in FLT3-ITD AML cell lines [150]. In vivo studies showed superior therapeutic efficacy of sitravatinib compared to gilteritinib in MOLM-13 xenograft and BaF3-FLT3-ITD models. In addition, sitravatinib maintained potent activity against FLT3 mutation in the presence of cytokines through the robust inhibition of p-ERK and p-AKT. Additionally, leukemic blasts of patients harboring the FLT3-ITD mutation were more sensitive to sitravatinib than gilteritinib in vitro and in a PDX model.

Sitravatinib is currently undergoing clinical evaluation as a monotherapy (NCT02978859) and in combination with other therapeutics like tislelizumab and nivolumab (NCT05407519, NCT04727996, NCT05228496, NCT03906071, NCT05542342, NCT04734262, NCT04887870 and NCT04904302) in different solid tumors.

### Rebastinib (DCC-2036; Deciphera Pharmaceuticals LLC)

Rebastinib, identified as a switch control inhibitor of BCR-ABL1 and FLT3 [151], was investigated in a phase 1 study in R/R CML and AML [152]. Although hematologic CR was achieved in 8/16 CML patients, including those with the T315I mutation, no responses were observed in AML patients. This insufficient clinical benefit led to the discontinuation of its development in both CML and AML. Pharmacodynamic analyses suggest that other kinases inhibited by rebastinib, such as TIE2, may be more relevant targets.

## AXL inhibitors in preclinical development

CTS2016 is a newly identified, selective, orally bioavailable small molecule inhibitor targeting AXL and FLT3 with single-digit nanomolar potency [153]. CTS2016 was evaluated both as a single agent and in combination with venetoclax or azacitidine in various in vitro and in vivo AML models and has been reported to exhibit strong growth inhibition and induced cell death in AML cell lines with FLT3 mutations. Combination treatments of CTS2016 with venetoclax or azacitidine showed enhanced therapeutic benefits compared to monotherapy, suggesting potential for use in R/R AML and MDS. Notably, CTS2016 effectively reduced tumor burden in a leukemia model with the drug-resistant FLT3-ITD-F691L mutation and displayed selectivity over other kinases, reducing off-target toxicities.

Niu et al.. discovered SLC-391, a potent inhibitor targeting MERTK, AXL, and TYRO3 with IC_50_ values of 44 nM, 9.6 nM, and 42.3 nM, respectively [64]. AML cell lines with high GAS6/AXL expression and MLL/FLT3-ITD mutations (MV4-11, MOLM-13), exhibited greater sensitivity to SLC-391 than cell lines with low GAS6/AXL expression and no MLL/FLT3-ITD mutations. Combining SLC-391 with venetoclax resulted in synergistic effects, significantly reducing cell viability, and increasing apoptosis in AML cell lines and primary patient cells, including stem cells, progenitors and myeloid blasts, as identified by multiple cell surface markers (e.g., CD47, CD44, CD99, CD123, and TIM3). In vivo, the combination therapy of SLC-391 and venetoclax delayed leukemia progression, prevented splenomegaly, and prolonged survival of both MV4-11 xenografted mice and AML PDX mice.

The novel MERTK and AXL inhibitor UNC569 [154, 155] has also shown promising preclinical efficacy. Koda, et al.. reported suppressed cell growth and induced apoptosis of OCI-AML5 and TMD7 AML cells upon UNC569 treatment, accompanied by reduced phosphorylation of MERTK, AKT and ERK and induced cleavage of caspase-3 [156].

### Anti-AXL antibodies

In 2019, Duan et al.. identified the AXL-targeting antibody DAXL-88, which effectively blocked the AXL-GAS6 interaction by binding with high affinity to both human and mouse AXL proteins [157, 158]. Additionally, DAXL-88 reversed the activation of key signaling molecules, including p-AXL, p-AKT, and p-ERK, which are typically upregulated by GAS6. Building on these findings, DAXL-88 was further developed into an antibody-drug conjugate (ADC), named DAXL-88-MMAE, by linking it with monomethyl auristatin E (MMAE), a cytotoxic agent that disrupts microtubule function. Upon binding to AXL, DAXL-88-MMAE is internalized, and the MMAE payload is released via lysosomal protease cleavage, leading to microtubule destabilization, cell cycle arrest, and apoptosis. In 2021, Liu et al.. extended the study of both DAXL-88 and DAXL-88-MMAE to FLT3-ITD^+^ AML, particularly in cells resistant to the FLT3 inhibitor quizartinib [159].

Their findings revealed that these AXL-targeted agents exhibited dose-dependent cytotoxicity in both FLT3-mutant AML cell lines (MV4-11 and quizartinib-resistant MV4-11) and primary AML blast cells from FLT3-ITD^+^ patients, especially when combined with quizartinib. The observed cytotoxicity is attributed to the inhibition of AXL, FLT3, and their downstream signaling pathways, which are crucial for the survival and proliferation of these cancer cells. Liu et al. further demonstrated that DAXL-88 could overcome resistance to the FLT3 inhibitors midostaurin and quizartinib, as well as to conventional chemotherapy agents used in the clinical ‘7 + 3’ induction regimen [159]. By generating chemo-resistant AML cell lines, it was showed that DAXL-88 exerts dose-dependent cytotoxicity in various AML models, including FLT3-WT THP-1, FLT3-ITD^+^ MV4-11, and quizartinib-resistant MV4-11 cells. This effect is likely mediated by blockade of the interaction between AXL and FLT3, thereby inhibiting downstream AKT/ERK signaling and inducing apoptosis. Notably, DAXL-88-MMAE demonstrated even stronger growth inhibition and pro-apoptotic effects in FLT3-ITD^+^ MV4-11 cells, accompanied by a reduction in both AXL and FLT3 signaling molecules, further enhancing its therapeutic potential.

The past years, we developed alpaca-derived, small sized sdAbs (12–15 kDa), cross-reactive for mouse and human AXL protein, and evaluated their potential for the diagnosis and treatment of AML [108]. We believe that sdAbs exhibit remarkable stability, solubility, and resistance to proteases, making them promising candidates for drug development with lower immunogenicity and simplified production processes compared to conventional mAbs [160, 161]. Several anti-AXL sdAbs were characterized using ELISA, flow cytometry, surface plasmon resonance and the AlphaFold2 prediction software. SdAb20 was selected as a lead compound for diagnosis and was, for therapeutic purposes, fused to a mouse IgG2a-Fc tail (sdAb20-Fc). Biodistribution studies demonstrated the tumor-specific uptake of sdAb20 in THP-1 xenografts and immunocompetent C1498 AML mice. Importantly, the signal in tumor-bearing mice was higher compared to the background signal in naive mice, illustrating its value as a diagnostic tracer in cancer patients. Therapeutically, we found that sdAb20-Fc demonstrated significant anti-tumor effects by inhibiting cell proliferation and cell viability in human AML cell lines and primary patient samples. Moreover, besides its clear single agent anti-tumor effect, our data also demonstrated the therapeutic potential of AXL-specific sdAb20-Fc in combination with the standard-of-care agents cytarabine and venetoclax. Interestingly, we found that cytarabine could induce AXL expression, and that the combination with sdAb20-Fc resulted in synergistic anti-AML effects. On the other hand, venetoclax had no effect on AXL expression and the combination with sdAb20-Fc resulted in additive effects in AML cell lines.

### Soluble receptors

Park et al.. reported that the inhibition of AXL activation using a soluble AXL chimeric protein, Axl-Fc, was able to diminish constitutive FLT3 phosphorylation in FLT3-ITD^+^ AML [109]. Furthermore, Axl-Fc disrupted the physical interaction between AXL and FLT3, inhibited cell growth, induced cell-cycle arrest and apoptosis, and alleviated the block in myeloid differentiation of FLT3-ITD^+^ AML cells in vitro. Later, it was even demonstrated that AXL inhibition with Axl-Fc substantially reduced resistance to the FLT3 inhibitors midostaurin and quizartinib using MOLM-13 FLT3-ITD^+^ AML cells [111].

Kariolis et al.. developed an AXL “decoy receptor” that binds GAS6 with high affinity and effectively inhibits its function [162]. This modified AXL variant contains four mutations, enhancing its affinity for GAS6 by 80-fold. The enhanced decoy receptor, MYD1-72, has been shown to significantly reduce cell growth and induce cytotoxicity in both OCI-AML3 and MV4-11 AML cells [163].

## Conclusion and future perspectives

The recognition of AXL as a critical mediator of AML pathogenesis has stimulated the development of AXL-targeted therapies aimed at disrupting AXL signaling and sensitizing leukemia cells to conventional chemotherapy. Small molecule inhibitors, monoclonal antibodies and sdAbs targeting AXL have shown promising preclinical efficacy in AML models, attenuating leukemia cell proliferation, inducing apoptosis, and enhancing the efficacy of standard chemotherapeutic agents. Additionally, combinatorial approaches with AXL inhibitors and targeted agents against other dysregulated pathways in AML, such as FLT3 inhibitors or BCL-2 inhibitors, hold potential for synergistic antileukemic effects and overcoming therapeutic resistance. In addition, recent studies also illustrated the importance of GAS6/AXL signaling in the immunosuppressive TME, fostering the evaluation of AXL-targeting therapies in combination with immunotherapies including immune checkpoint inhibitors.

Despite the therapeutic promise of targeting AXL in AML, several challenges remain to be addressed. Resistance mechanisms, including acquired mutations in AXL or activation of compensatory signaling pathways, can limit the efficacy of AXL-targeted therapies, necessitating the development of next-generation inhibitors or rational drug combinations. Furthermore, the identification of predictive biomarkers to stratify AML patients who are most likely to benefit from AXL-targeted therapies is imperative for optimizing treatment selection and improving clinical outcomes. We believe that sdAbs, with their unique advantages over conventional mAbs, are promising candidates for drug development with lower immunogenicity and simplified production processes compared to conventional mAb [108].

The potential toxicity and off-target effects associated with systemic AXL inhibition underscore the need for targeted delivery strategies to minimize adverse effects while maximizing therapeutic efficacy. In conclusion, AXL represents a promising therapeutic target in AML, given its pivotal role in disease pathogenesis, adverse prognostic implications, and therapeutic vulnerabilities. Elucidating the mechanisms driving AXL dysregulation and the development of AXL-targeted therapies, especially in combinatorial regimens with chemo- and immunotherapy, hold great promise for improving the outcomes of AML patients, particularly those with high-risk disease features and refractory disease. Continued research efforts aimed at unraveling the complexities of AXL signaling and translating these findings into clinical practice are required to reveal the full therapeutic potential of AXL targeting in AML.

## Data Availability

No datasets were generated or analysed during the current study.

## Abbreviations

ADC: Antibody-drug conjugates
ALK: Anaplastic lymphoma kinase
AML: Acute myeloid leukemia
BCL-2: B-cell lymphoma 2
BM: Bone marrow
BMSC: Bone marrow stromal cells
CML: Chronic myeloid leukemia
CR: Complete remission
DC: Dendritic cell
ECD: Extracellular domain
ECM: Extracellular matrix
EGF: Epidermal growth factor receptor
EMT: Epithelial-to-mesenchymal transition
ERK: Extracellular signal-regulated kinase
FDA: Food and Drug Administration
FLT3: Fms-like tyrosine kinase-3
GAS6: Growth-arrest specific protein 6
HSC: Hematopoietic stem cell
IDH: Isocitrate dehydrogenase
IFN: Interferon
Ig: Immunoglobulin
IL: Interleukin
ITD: Internal tandem duplication
JAK: Janus kinase
KO: Knockout
LSCs: Leukemic stem cells
MAPK: Mitogen-activated protein kinase
MCL-1: Myeloid cell leukemia-1
MDS: Myelodysplastic syndrome
MDSC: Myeloid-derived suppressor cell
MHC-I or –II: MHC class I or II
MLL: Mixed lineage leukemia
NFκB: Nuclear factor kappa-light-chain-enhancer of activated B cells
NK cell: Natural killer cell
NPM1: Nucleophosmin 1
NSCLC: Non-small cell lung carcinoma
OS: Overall survival
PDGFR: Platelet-derived growth factor receptor
PI3K: Phosphatidylinositol 3-kinase
R/R: Relapsed/refractory
RCC: Renal cell carcinoma
RON: Recepteur d’origine nantais
RTK: Receptor tyrosine kinase
STAT: Signal transducer and activator of transcription
TKI: Tyrosine kinase inhibitor
Tregs: Regulatory T cells
TGF-β: Transforming growth factor beta
TME: Tumor microenvironment
TNF: Tumor necrosis factor
VEGFR: Vascular endothelial growth factor receptor

## References

[1] Vakiti A, Mewawalla P. *Acute myeloid leukemia*, in *StatPearls*. 2024: Treasure Island (FL) ineligible companies. Disclosure: Prerna Mewawalla declares no relevant financial relationships with ineligible companies.

[2] Blanco MNF, Belderbos M, Vormoor HJ. Leukemia suppressing normal bone marrow: how long does it last? Haematologica. 2023;108(11):2891–3. 37021524 10.3324/haematol.2023.282955 PMC10620553

[3] Abdallah M, et al. Older patients’ experiences following initial diagnosis of acute myeloid leukemia: a qualitative study. J Geriatr Oncol. 2022;13(8):1230–5. 36064536 10.1016/j.jgo.2022.08.017 PMC9982634

[4] De Kouchkovsky I, Abdul-Hay M. Acute myeloid leukemia: a comprehensive review and 2016 update. Blood Cancer J. 2016;6(7):e441. 27367478 10.1038/bcj.2016.50 PMC5030376

[5] DiNardo CD, Cortes JE. Mutations in AML: prognostic and therapeutic implications. Hematol Am Soc Hematol Educ Program. 2016;2016(1):348–55. 10.1182/asheducation-2016.1.348 PMC6142505 27913501

[6] Stirewalt DL, Radich JP. The role of FLT3 in haematopoietic malignancies. Nat Rev Cancer. 2003;3(9):650–65. 12951584 10.1038/nrc1169

[7] Knapper S. FLT3 inhibition in acute myeloid leukaemia. Br J Haematol. 2007;138(6):687–99. 17655729 10.1111/j.1365-2141.2007.06700.x

[8] Issa GC, et al. Clinical outcomes associated with NPM1 mutations in patients with relapsed or refractory AML. Blood Adv. 2023;7(6):933–42. 36322818 10.1182/bloodadvances.2022008316 PMC10027507

[9] Daver N, et al. New directions for emerging therapies in acute myeloid leukemia: the next chapter. Blood Cancer J. 2020;10(10):107. 33127875 10.1038/s41408-020-00376-1 PMC7599225

[10] Roman Diaz JL, Martinez MV, Khimani F. New approaches for the treatment of AML beyond the 7 + 3 regimen: current concepts and New approaches. Volume 16. Cancers (Basel); 2024. 3. 10.3390/cancers16030677 PMC10854755 38339429

[11] Whitman SP, et al. GAS6 expression identifies high-risk adult AML patients: potential implications for therapy. Leukemia. 2014;28(6):1252–8. 24326683 10.1038/leu.2013.371 PMC4047202

[12] Ben-Batalla I, et al. Axl, a prognostic and therapeutic target in acute myeloid leukemia mediates paracrine crosstalk of leukemia cells with bone marrow stroma. Blood. 2013;122(14):2443–52. 23982172 10.1182/blood-2013-03-491431

[13] Yan S, et al. AXL Receptor Tyrosine Kinase as a therapeutic target in hematological malignancies: focus on multiple myeloma. Cancers (Basel). 2019;11(11):1727. 31694201 10.3390/cancers11111727 PMC6896070

[14] O’Bryan JP, et al. Axl, a transforming gene isolated from primary human myeloid leukemia cells, encodes a novel receptor tyrosine kinase. Mol Cell Biol. 1991;11(10):5016–31. 1656220 10.1128/mcb.11.10.5016-5031.1991 PMC361494

[15] Caberoy NB, et al. Galectin-3 is a new MerTK-specific eat-me signal. J Cell Physiol. 2012;227(2):401–7. 21792939 10.1002/jcp.22955 PMC3225605

[16] Caberoy NB, Zhou Y, Li W. Tubby and Tubby-like protein 1 are new MerTK ligands for phagocytosis. EMBO J. 2010;29(23):3898–910. 20978472 10.1038/emboj.2010.265 PMC3020645

[17] Hafizi S, Dahlback B. Gas6 and protein S. vitamin K-dependent ligands for the Axl receptor tyrosine kinase subfamily. FEBS J. 2006;273(23):5231–44. 17064312 10.1111/j.1742-4658.2006.05529.x

[18] Lemke G, Rothlin CV. Immunobiology of the TAM receptors. Nat Rev Immunol. 2008;8(5):327–36. 18421305 10.1038/nri2303 PMC2856445

[19] Stitt TN, et al. The anticoagulation factor protein S and its relative, Gas6, are ligands for the Tyro 3/Axl family of receptor tyrosine kinases. Cell. 1995;80(4):661–70. 7867073 10.1016/0092-8674(95)90520-0

[20] Ohashi K, et al. Stimulation of sky receptor tyrosine kinase by the product of growth arrest-specific gene 6. J Biol Chem. 1995;270(39):22681–4. 7559388 10.1074/jbc.270.39.22681

[21] Sasaki T, et al. Structural basis for Gas6-Axl signalling. EMBO J. 2006;25(1):80–7. 16362042 10.1038/sj.emboj.7600912 PMC1356355

[22] Nagata K, et al. Identification of the product of growth arrest-specific gene 6 as a common ligand for Axl, Sky, and mer receptor tyrosine kinases. J Biol Chem. 1996;271(47):30022–7. 8939948 10.1074/jbc.271.47.30022

[23] Scaltriti M, Elkabets M, Baselga J. Molecular pathways: AXL, a membrane receptor mediator of resistance to Therapy. Clin Cancer Res. 2016;22(6):1313–7. 26763248 10.1158/1078-0432.CCR-15-1458 PMC4957976

[24] Varnum BC, et al. Axl receptor tyrosine kinase stimulated by the vitamin K-dependent protein encoded by growth-arrest-specific gene 6. Nature. 1995;373(6515):623–6. 7854420 10.1038/373623a0

[25] Schneider C, King RM, Philipson L. Genes specifically expressed at growth arrest of mammalian cells. Cell. 1988;54(6):787–93. 3409319 10.1016/s0092-8674(88)91065-3

[26] Dagamajalu S, et al. A pathway map of AXL receptor-mediated signaling network. J Cell Commun Signal. 2021;15(1):143–8. 32829427 10.1007/s12079-020-00580-5 PMC7905004

[27] Fujimori T, et al. The axl receptor tyrosine kinase is a discriminator of macrophage function in the inflamed lung. Mucosal Immunol. 2015;8(5):1021–30. 25603826 10.1038/mi.2014.129 PMC4430298

[28] Fridell YW, et al. Differential activation of the Ras/extracellular-signal-regulated protein kinase pathway is responsible for the biological consequences induced by the Axl receptor tyrosine kinase. Mol Cell Biol. 1996;16(1):135–45. 8524290 10.1128/mcb.16.1.135 PMC230987

[29] Bellosta P, et al. The receptor tyrosine kinase ARK mediates cell aggregation by homophilic binding. Mol Cell Biol. 1995;15(2):614–25. 7823930 10.1128/mcb.15.2.614 PMC231916

[30] Burchert A, et al. Determinants for transformation induced by the Axl receptor tyrosine kinase. Oncogene. 1998;16(24):3177–87. 9671397 10.1038/sj.onc.1201865

[31] Konishi A, et al. Hydrogen peroxide activates the Gas6-Axl pathway in vascular smooth muscle cells. J Biol Chem. 2004;279(27):28766–70. 15123721 10.1074/jbc.M401977200

[32] Pierce A, et al. Axl and Tyro3 modulate female reproduction by influencing gonadotropin-releasing hormone neuron survival and migration. Mol Endocrinol. 2008;22(11):2481–95. 18787040 10.1210/me.2008-0169 PMC2582545

[33] Brown JE, et al. Cross-phosphorylation, signaling and proliferative functions of the Tyro3 and Axl receptors in Rat2 cells. PLoS ONE. 2012;7(5):e36800. 22606290 10.1371/journal.pone.0036800 PMC3351477

[34] Meyer AS, et al. The receptor AXL diversifies EGFR signaling and limits the response to EGFR-targeted inhibitors in triple-negative breast cancer cells. Sci Signal. 2013;6(287):ra66. 23921085 10.1126/scisignal.2004155 PMC3947921

[35] Vouri M, et al. Axl-EGFR receptor tyrosine kinase hetero-interaction provides EGFR with access to pro-invasive signalling in cancer cells. Oncogenesis. 2016;5(10):e266. 27775700 10.1038/oncsis.2016.66 PMC5117851

[36] Salian-Mehta S, Xu M, Wierman ME. AXL and MET crosstalk to promote gonadotropin releasing hormone (GnRH) neuronal cell migration and survival. Mol Cell Endocrinol. 2013;374(1–2):92–100. 23648337 10.1016/j.mce.2013.04.018 PMC3690482

[37] Ruan GX, Kazlauskas A. Axl is essential for VEGF-A-dependent activation of PI3K/Akt. EMBO J. 2012;31(7):1692–703. 22327215 10.1038/emboj.2012.21 PMC3321201

[38] Goyette MA, Cote JF. AXL Receptor Tyrosine Kinase as a Promising Therapeutic Target directing multiple aspects of Cancer Progression and Metastasis. Cancers (Basel), 2022. 14(3). 10.3390/cancers14030466 PMC8833413 35158733

[39] Shen Y, et al. Axl inhibitors as novel cancer therapeutic agents. Life Sci. 2018;198:99–111. 29496493 10.1016/j.lfs.2018.02.033

[40] Wu X et al. Quantitative tyrosine phosphoproteome profiling of AXL Receptor Tyrosine Kinase Signaling Network. Cancers (Basel), 2021. 13(16). 10.3390/cancers13164234 PMC8394654 34439388

[41] Lauter M, Weber A, Torka R. Targeting of the AXL receptor tyrosine kinase by small molecule inhibitor leads to AXL cell surface accumulation by impairing the ubiquitin-dependent receptor degradation. Cell Commun Signal. 2019;17(1):59. 31171001 10.1186/s12964-019-0377-8 PMC6555758

[42] Huang H. *Proteolytic Cleavage of Receptor Tyrosine Kinases.* Biomolecules, 2021. 11(5). 10.3390/biom11050660 PMC8145142 33947097

[43] Tanaka M, Siemann DW. Gas6/Axl Signaling Pathway in the Tumor Immune Microenvironment. Cancers (Basel), 2020. 12(7). 10.3390/cancers12071850 PMC7408754 32660000

[44] Kim EM, et al. Axl signaling induces development of natural killer cells in vitro and in vivo. Protoplasma. 2017;254(2):1091–101. 27549806 10.1007/s00709-016-1016-5

[45] Grabiec AM, et al. Axl and MerTK receptor tyrosine kinases maintain human macrophage efferocytic capacity in the presence of viral triggers. Eur J Immunol. 2018;48(5):855–60. 29400409 10.1002/eji.201747283 PMC6001567

[46] Villani AC et al. Single-cell RNA-seq reveals new types of human blood dendritic cells, monocytes, and progenitors. Science, 2017. 356(6335). 10.1126/science.aah4573 PMC5775029 28428369

[47] Gjerdrum C, et al. Axl is an essential epithelial-to-mesenchymal transition-induced regulator of breast cancer metastasis and patient survival. Proc Natl Acad Sci U S A. 2010;107(3):1124–9. 20080645 10.1073/pnas.0909333107 PMC2824310

[48] Gustafsson A, et al. Differential expression of Axl and Gas6 in renal cell carcinoma reflecting tumor advancement and survival. Clin Cancer Res. 2009;15(14):4742–9. 19567592 10.1158/1078-0432.CCR-08-2514

[49] Hutterer M, et al. Axl and growth arrest-specific gene 6 are frequently overexpressed in human gliomas and predict poor prognosis in patients with glioblastoma multiforme. Clin Cancer Res. 2008;14(1):130–8. 18172262 10.1158/1078-0432.CCR-07-0862

[50] Shieh YS, et al. Expression of axl in lung adenocarcinoma and correlation with tumor progression. Neoplasia. 2005;7(12):1058–64. 16354588 10.1593/neo.05640 PMC1501169

[51] Tang Y, et al. AXL in cancer: a modulator of drug resistance and therapeutic target. J Exp Clin Cancer Res. 2023;42(1):148. 37328828 10.1186/s13046-023-02726-w PMC10273696

[52] D’Alfonso TM, et al. Axl receptor tyrosine kinase expression in breast cancer. J Clin Pathol. 2014;67(8):690–6. 24904064 10.1136/jclinpath-2013-202161 PMC4549806

[53] Ozyurt R, Ozpolat B. Therapeutic Landscape of AXL receptor kinase in Triple-negative breast Cancer. Mol Cancer Ther. 2023;22(7):818–32. 37028809 10.1158/1535-7163.MCT-22-0617

[54] Nonagase Y, et al. Tumor tissue and plasma levels of AXL and GAS6 before and after tyrosine kinase inhibitor treatment in EGFR-mutated non-small cell lung cancer. Thorac Cancer. 2019;10(10):1928–35. 31419057 10.1111/1759-7714.13166 PMC6775020

[55] Zhang G, et al. Function of Axl receptor tyrosine kinase in non-small cell lung cancer. Oncol Lett. 2018;15(3):2726–34. 29434997 10.3892/ol.2017.7694 PMC5778882

[56] Sang YB, et al. The development of AXL inhibitors in Lung Cancer: recent Progress and challenges. Front Oncol. 2022;12:811247. 35311091 10.3389/fonc.2022.811247 PMC8927964

[57] Martinelli E, et al. AXL is an oncotarget in human colorectal cancer. Oncotarget. 2015;6(27):23281–96. 25966280 10.18632/oncotarget.3962 PMC4695118

[58] Lozneanu L, et al. Computational and immunohistochemical analyses highlight AXL as a potential prognostic marker for ovarian Cancer patients. Anticancer Res. 2016;36(8):4155–63. 27466525

[59] Du W, et al. AXL is a key factor for cell plasticity and promotes metastasis in pancreatic Cancer. Mol Cancer Res. 2021;19(8):1412–21. 33811159 10.1158/1541-7786.MCR-20-0860 PMC8349827

[60] Paccez JD, et al. The receptor tyrosine kinase Ax1 is an essential regulator of prostate cancer proliferation and tumor growth and represents a new therapeutic target. Oncogene. 2013;32(6):689–98. 22410775 10.1038/onc.2012.89 PMC4078100

[61] Sensi M, et al. Human cutaneous melanomas lacking MITF and Melanocyte Differentiation Antigens Express a functional axl receptor kinase. J Invest Dermatology. 2011;131(12):2448–57. 10.1038/jid.2011.218 21796150

[62] Rochlitz C, et al. Axl expression is associated with adverse prognosis and with expression of Bcl-2 and CD34 in de novo acute myeloid leukemia (AML): results from a multicenter trial of the Swiss Group for Clinical Cancer Research (SAKK). Leukemia. 1999;13(9):1352–8. 10482985 10.1038/sj.leu.2401484

[63] Fatima M, et al. AXL receptor tyrosine kinase: a possible therapeutic target in acute promyelocytic leukemia. BMC Cancer. 2021;21(1):713. 34140003 10.1186/s12885-021-08450-y PMC8210361

[64] Niu X, et al. Targeting AXL kinase sensitizes leukemic stem and progenitor cells to venetoclax treatment in acute myeloid leukemia. Blood. 2021;137(26):3641–55. 33786587 10.1182/blood.2020007651 PMC8462401

[65] Gay CM, Balaji K, Byers LA. Giving AXL the axe: targeting AXL in human malignancy. Br J Cancer. 2017;116(4):415–23. 28072762 10.1038/bjc.2016.428 PMC5318970

[66] Axelrod H, Pienta KJ. Axl as a mediator of cellular growth and survival. Oncotarget. 2014;5(19):1–35. 25344858 10.18632/oncotarget.2422 PMC4253401

[67] Zhu C, Wei Y, Wei X. AXL receptor tyrosine kinase as a promising anti-cancer approach: functions, molecular mechanisms and clinical applications. Mol Cancer. 2019;18(1):153. 31684958 10.1186/s12943-019-1090-3 PMC6827209

[68] Linger RMA, et al. TAM receptor tyrosine kinases: biologic functions, signaling, and potential therapeutic targeting in human cancer. Adv Cancer Res. 2008;100:35–. 18620092 10.1016/S0065-230X(08)00002-X PMC3133732

[69] May CD, et al. AXL is a potential therapeutic target in dedifferentiated and pleomorphic liposarcomas. BMC Cancer. 2015;15:901. 26573603 10.1186/s12885-015-1916-3 PMC4647521

[70] Corno C, et al. Role of the Receptor Tyrosine Kinase Axl and its Targeting in Cancer cells. Curr Med Chem. 2016;23(15):1496–512. 27048336 10.2174/0929867323666160405112954

[71] Hasanbasic I, et al. Intracellular signaling pathways involved in Gas6-Axl-mediated survival of endothelial cells. Am J Physiol Heart Circ Physiol. 2004;287(3):H1207–13. 15130893 10.1152/ajpheart.00020.2004

[72] Hong CC, et al. Receptor tyrosine kinase AXL is induced by chemotherapy drugs and overexpression of AXL confers drug resistance in acute myeloid leukemia. Cancer Lett. 2008;268(2):314–24. 18502572 10.1016/j.canlet.2008.04.017

[73] Zdzalik-Bielecka D et al. The GAS6-AXL signaling pathway triggers actin remodeling that drives membrane ruffling, macropinocytosis, and cancer-cell invasion. Proc Natl Acad Sci U S A, 2021. 118(28). 10.1073/pnas.2024596118 PMC8285903 34244439

[74] Abu-Thuraia A, et al. AXL confers cell migration and invasion by hijacking a PEAK1-regulated focal adhesion protein network. Nat Commun. 2020;11(1):3586. 32681075 10.1038/s41467-020-17415-x PMC7368075

[75] Maacha S, et al. AXL mediates esophageal Adenocarcinoma Cell Invasion through Regulation of Extracellular acidification and lysosome trafficking. Neoplasia. 2018;20(10):1008–22. 30189359 10.1016/j.neo.2018.08.005 PMC6126204

[76] Niland S, Riscanevo AX, Eble JA. Matrix metalloproteinases shape the Tumor Microenvironment in Cancer Progression. Int J Mol Sci, 2021. 23(1). 10.3390/ijms23010146 PMC8745566 35008569

[77] Koorstra JB, et al. The axl receptor tyrosine kinase confers an adverse prognostic influence in pancreatic cancer and represents a new therapeutic target. Cancer Biol Ther. 2009;8(7):618–26. 19252414 10.4161/cbt.8.7.7923 PMC2678175

[78] Antony J, Huang RY. AXL-Driven EMT state as a Targetable Conduit in Cancer. Cancer Res. 2017;77(14):3725–32. 28667075 10.1158/0008-5472.CAN-17-0392

[79] Tanaka M, Siemann DW. Axl signaling is an important mediator of tumor angiogenesis. Oncotarget. 2019;10(30):2887–98. 31080559 10.18632/oncotarget.26882 PMC6499597

[80] Ruan GX, Kazlauskas A. Lactate engages receptor tyrosine kinases Axl, Tie2, and vascular endothelial growth factor receptor 2 to activate phosphoinositide 3-kinase/Akt and promote angiogenesis. J Biol Chem. 2013;288(29):21161–72. 23754286 10.1074/jbc.M113.474619 PMC3774382

[81] Holtzhausen A, et al. TAM Family receptor kinase inhibition reverses MDSC-Mediated suppression and augments Anti-PD-1 therapy in Melanoma. Cancer Immunol Res. 2019;7(10):1672–86. 31451482 10.1158/2326-6066.CIR-19-0008 PMC6943983

[82] Chiu KC, et al. Polarization of tumor-associated macrophages and Gas6/Axl signaling in oral squamous cell carcinoma. Oral Oncol. 2015;51(7):683–9. 25910588 10.1016/j.oraloncology.2015.04.004

[83] Myers KV, Amend SR, Pienta KJ. Targeting Tyro3, Axl and MerTK (TAM receptors): implications for macrophages in the tumor microenvironment. Mol Cancer. 2019;18(1):94. 31088471 10.1186/s12943-019-1022-2 PMC6515593

[84] Wu G, et al. Targeting Gas6/TAM in cancer cells and tumor microenvironment. Mol Cancer. 2018;17(1):20. 29386018 10.1186/s12943-018-0769-1 PMC5793417

[85] Aehnlich P et al. TAM receptor inhibition-implications for Cancer and the Immune System. Cancers (Basel), 2021. 13(6). 10.3390/cancers13061195 PMC7998716 33801886

[86] Lai S, et al. Abstract B148: activity of the TAM kinase-targeting compound, SLC-391, is mediated by the engagement of the immune system in CT-26 syngeneic mouse model. Mol Cancer Ther. 2018;17:B148–148.

[87] Goyette MA, et al. Targeting Axl favors an antitumorigenic microenvironment that enhances immunotherapy responses by decreasing Hif-1alpha levels. Volume 118. Proc Natl Acad Sci U S A; 2021. 29. 10.1073/pnas.2023868118 PMC8307381 34266948

[88] Paolino M, et al. The E3 ligase Cbl-b and TAM receptors regulate cancer metastasis via natural killer cells. Nature. 2014;507(7493):508–12. 24553136 10.1038/nature12998 PMC6258903

[89] Terry S, et al. AXL Targeting overcomes human Lung Cancer Cell Resistance to NK- and CTL-Mediated cytotoxicity. Cancer Immunol Res. 2019;7(11):1789–802. 31488404 10.1158/2326-6066.CIR-18-0903

[90] Zhao GJ, et al. Growth arrest-specific 6 enhances the suppressive function of CD4(+) CD25(+) Regulatory T cells mainly through Axl Receptor. Mediators of Inflammation; 2017. 10.1155/2017/6848430 PMC5320320 28270700

[91] Aguilera TA et al. Reprogramming the immunological microenvironment through radiation and targeting Axl. Nat Commun, 2016. 7. 10.1038/ncomms13898 PMC5196438 28008921

[92] Kasikara C, et al. Phosphatidylserine sensing by TAM receptors regulates AKT-Dependent chemoresistance and PD-L1 expression. Mol Cancer Res. 2017;15(6):753–64. 28184013 10.1158/1541-7786.MCR-16-0350 PMC8363069

[93] Boshuizen J, et al. Cooperative Targeting of Immunotherapy-Resistant Melanoma and Lung Cancer by an AXL-Targeting antibody-drug Conjugate and Immune Checkpoint Blockade. Cancer Res. 2021;81(7):1775–87. 33531370 10.1158/0008-5472.CAN-20-0434

[94] Sadahiro H, et al. Activation of the Receptor Tyrosine Kinase AXL regulates the Immune Microenvironment in Glioblastoma. Cancer Res. 2018;78(11):3002–13. 29531161 10.1158/0008-5472.CAN-17-2433 PMC5984695

[95] Lee JH, et al. Transcriptional downregulation of MHC class I and melanoma de- differentiation in resistance to PD-1 inhibition. Nat Commun. 2020;11(1):1897. 32312968 10.1038/s41467-020-15726-7 PMC7171183

[96] Chen XH, et al. TGF-beta and EGF induced HLA-I downregulation is associated with epithelial-mesenchymal transition (EMT) through upregulation of snail in prostate cancer cells. Mol Immunol. 2015;65(1):34–42. 25618241 10.1016/j.molimm.2014.12.017

[97] Terry S, et al. Abstract 5754: hypoxia-induced tumor plasticity and immune resistance involves an alteration of target recognition by a mechanism involving TGF-beta signaling. Cancer Res. 2018;78(13Supplement):5754–5754.30185548

[98] Neubauer A, et al. Expression of axl, a transforming receptor tyrosine kinase, in normal and malignant hematopoiesis. Blood. 1994;84(6):1931–41. 7521695

[99] Yang X et al. ssExpression level of GAS6-mRNA influences the prognosis of acute myeloid leukemia patients with allogeneic hematopoietic stem cell transplantation. Biosci Rep, 2019. 39(5). 10.1042/BSR20190389 PMC6527924 31028135

[100] Tirado-Gonzalez I, et al. AXL Inhibition in Macrophages stimulates host-versus-leukemia immunity and eradicates naive and treatment-resistant leukemia. Cancer Discov. 2021;11(11):2924–43. 34103328 10.1158/2159-8290.CD-20-1378 PMC7611942

[101] Wang J, et al. Leukemogenic chromatin alterations promote AML Leukemia Stem cells via a KDM4C-ALKBH5-AXL Signaling Axis. Cell Stem Cell. 2020;27(1):81–e978. 32402251 10.1016/j.stem.2020.04.001

[102] Lin JZ, et al. Targeting AXL overcomes resistance to docetaxel therapy in advanced prostate cancer. Oncotarget. 2017;8(25):41064–77. 28455956 10.18632/oncotarget.17026 PMC5522277

[103] Brand TM, et al. AXL is a logical Molecular Target in Head and Neck squamous cell carcinoma. Clin Cancer Res. 2015;21(11):2601–12. 25767293 10.1158/1078-0432.CCR-14-2648 PMC5032632

[104] Hugo W, et al. Genomic and transcriptomic features of response to Anti-PD-1 therapy in metastatic melanoma. Cell. 2016;165(1):35–44. 26997480 10.1016/j.cell.2016.02.065 PMC4808437

[105] Giles KM, et al. Axl mediates acquired resistance of head and neck cancer cells to the epidermal growth factor receptor inhibitor erlotinib. Mol Cancer Ther. 2013;12(11):2541–58. 24026012 10.1158/1535-7163.MCT-13-0170

[106] Tian Y, et al. Anexelekto (AXL) increases resistance to EGFR-TKI and activation of AKT and ERK1/2 in Non-small Cell Lung Cancer cells. Oncol Res. 2016;24(5):295–303. 27712586 10.3727/096504016X14648701447814 PMC7838623

[107] Mahadevan D, et al. A novel tyrosine kinase switch is a mechanism of imatinib resistance in gastrointestinal stromal tumors. Oncogene. 2007;26(27):3909–19. 17325667 10.1038/sj.onc.1210173

[108] Vandewalle N, et al. AXL-specific single domain antibodies show diagnostic potential and anti-tumor activity in Acute myeloid leukemia. Theranostics. 2024;14(7):2656–74. 38773967 10.7150/thno.91456 PMC11103505

[109] Park IK, et al. Inhibition of the receptor tyrosine kinase Axl impedes activation of the FLT3 internal tandem duplication in human acute myeloid leukemia: implications for Axl as a potential therapeutic target. Blood. 2013;121(11):2064–73. 23321254 10.1182/blood-2012-07-444018 PMC3596966

[110] Park IK, et al. The Axl/Gas6 pathway is required for optimal cytokine signaling during human natural killer cell development. Blood. 2009;113(11):2470–7. 18840707 10.1182/blood-2008-05-157073 PMC2656272

[111] Park IK, et al. Receptor tyrosine kinase Axl is required for resistance of leukemic cells to FLT3-targeted therapy in acute myeloid leukemia. Leukemia. 2015;29(12):2382–9. 26172401 10.1038/leu.2015.147 PMC8145983

[112] Dumas PY, et al. Hematopoietic niche drives FLT3-ITD acute myeloid leukemia resistance to quizartinib via STAT5-and hypoxia-dependent upregulation of AXL. Haematologica. 2019;104(10):2017–27. 30923103 10.3324/haematol.2018.205385 PMC6886433

[113] Post SM, et al. AXL/MERTK inhibitor ONO-7475 potently synergizes with venetoclax and overcomes venetoclax resistance to kill FLT3-ITD acute myeloid leukemia. Haematologica. 2022;107(6):1311–22. 34732043 10.3324/haematol.2021.278369 PMC9152975

[114] Myers SH, Brunton VG, Unciti-Broceta A. AXL inhibitors in Cancer: a Medicinal Chemistry Perspective. J Med Chem. 2016;59(8):3593–608. 26555154 10.1021/acs.jmedchem.5b01273

[115] Zhao Z, et al. Exploration of type II binding mode: a privileged approach for kinase inhibitor focused drug discovery? ACS Chem Biol. 2014;9(6):1230–41. 24730530 10.1021/cb500129t PMC4068218

[116] Usuki K, et al. Clinical profile of gilteritinib in Japanese patients with relapsed/refractory acute myeloid leukemia: an open-label phase 1 study. Cancer Sci. 2018;109(10):3235–44. 30039554 10.1111/cas.13749 PMC6172068

[117] Loges S, et al. A first-in-patient phase I study of BGB324, a selective Axl kinase inhibitor in patients with refractory/relapsed AML and high-risk MDS. J Clin Oncol. 2016;34(15suppl):2561–2561.27185848

[118] Mims AS, et al. A phase 1b/2 study of TP-0903 and decitabine targeting mutant TP53 and/or complex karyotype in patients with untreated acute myeloid leukemia ≥ age 60 years: phase 1b interim results. J Clin Oncol. 2022;40(16suppl):7027–7027.

[119] Sheridan C. First Axl inhibitor enters clinical trials. Nat Biotechnol. 2013;31(9):775–6. 24022140 10.1038/nbt0913-775a

[120] Holland SJ, et al. R428, a selective small molecule inhibitor of Axl Kinase, blocks Tumor Spread and Prolongs Survival in models of metastatic breast Cancer. Cancer Res. 2010;70(4):1544–54. 20145120 10.1158/0008-5472.CAN-09-2997

[121] Chen FF, Song QL, Yu Q. Axl inhibitor R428 induces apoptosis of cancer cells by blocking lysosomal acidification and recycling independent of Axl inhibition. Am J Cancer Res. 2018;8(8):1466–. 30210917 PMC6129480

[122] Loges S et al. A first-in-patient phase I study of BGB324, a selective Axl kinase inhibitor in patients with refractory/relapsed AML and high-risk MDS. J Clin Oncol, 2016. 34(15).

[123] Loges S, et al. Phase Ib/II study (NCT02488408 / BGBC003) of Bemcentinib Monotherapy or in combination with cytarabine or decitabine in Acute myeloid leukemia (AML) or myelodysplastic syndrome (MDS): FINAL results. Blood. 2023;142(Supplement 1):4287–4287.

[124] Beumer N, et al. AML Treatment by the AXL inhibitor Bemcentinib in Combination with Cytarabine shows clinical efficacy related to TNFα and cytotoxic Immune cells: a single-cell translational study from the BGBC003 trial. Blood. 2023;142(Supplement 1):1540–1540.

[125] Kubasch AS, et al. Efficacy and safety of bemcentinib in patients with advanced myelodysplastic neoplasms or acute myeloid leukemia failing hypomethylating agents- the EMSCO phase II BERGAMO trial. Leukemia. 2023;37(11):2309–13. 37735558 10.1038/s41375-023-02029-1 PMC10624604

[126] Mollard A, et al. Design, synthesis and biological evaluation of a series of novel axl kinase inhibitors. ACS Med Chem Lett. 2011;2(12):907–12. 22247788 10.1021/ml200198x PMC3254106

[127] Jimenez L, et al. Phenotypic chemical screening using a zebrafish neural crest EMT reporter identifies retinoic acid as an inhibitor of epithelial morphogenesis. Dis Model Mech. 2016;9(4):389–400. 26794130 10.1242/dmm.021790 PMC4852498

[128] Sinha S, et al. Targeted Axl Inhibition primes chronic lymphocytic leukemia B cells to apoptosis and shows Synergistic/Additive effects in Combination with BTK inhibitors. Clin Cancer Res. 2015;21(9):2115–26. 25673699 10.1158/1078-0432.CCR-14-1892 PMC4479154

[129] Patel V, et al. Preclinical combination of TP-0903, an AXL inhibitor and B-PAC-1, a procaspase-activating compound with ibrutinib in chronic lymphocytic leukemia. Leuk Lymphoma. 2016;57(6):1494–7. 26440741 10.3109/10428194.2015.1102243 PMC4963009

[130] Jeon JY et al. TP-0903, a novel axl inhibitor with activity in drug resistant FLT3-ITD + AML through a mechanism that includes FLT3 inhibition. Blood. 2017;130(Supplement 1):2522.

[131] Jeon JY et al. TP-0903 is active in models of drug-resistant acute myeloid leukemia. JCI Insight, 2020. 5(23). 10.1172/jci.insight.140169 PMC7714403 33268594

[132] Eisenmann ED et al. TP-0903 is active in preclinical models of Acute myeloid leukemia with TP53 Mutation/Deletion. Cancers (Basel), 2022. 15(1). 10.3390/cancers15010029 PMC9817780 36612026

[133] Mori M, et al. Gilteritinib, a FLT3/AXL inhibitor, shows antileukemic activity in mouse models of FLT3 mutated acute myeloid leukemia. Invest New Drugs. 2017;35(5):556–65. 28516360 10.1007/s10637-017-0470-z PMC5613053

[134] Dumas PY, et al. Dual inhibition of FLT3 and AXL by Gilteritinib overcomes hematopoietic niche-driven resistance mechanisms in FLT3-ITD Acute myeloid leukemia. Clin Cancer Res. 2021;27(21):6012–25. 34400415 10.1158/1078-0432.CCR-20-3114

[135] Perl AE, et al. Selective inhibition of FLT3 by gilteritinib in relapsed or refractory acute myeloid leukaemia: a multicentre, first-in-human, open-label, phase 1–2 study. Lancet Oncol. 2017;18(8):1061–75. 28645776 10.1016/S1470-2045(17)30416-3 PMC5572576

[136] Perl AE, et al. Clinical outcomes in patients with relapsed/refractory FLT3-mutated acute myeloid leukemia treated with gilteritinib who received prior midostaurin or sorafenib. Blood Cancer J. 2022;12(5):84. 35637252 10.1038/s41408-022-00677-7 PMC9151663

[137] Perl AE, et al. Gilteritinib or Chemotherapy for relapsed or refractory FLT3-Mutated AML. N Engl J Med. 2019;381(18):1728–40. 31665578 10.1056/NEJMoa1902688

[138] Daver N, et al. Venetoclax Plus Gilteritinib for FLT3-Mutated Relapsed/Refractory Acute Myeloid Leukemia. J Clin Oncol. 2022;40(35):4048–59. 35849791 10.1200/JCO.22.00602 PMC9746764

[139] Altman JK, et al. Gilteritinib can be safely combined with Atezolizumab for the treatment of relapsed or refractory FLT3-Mutated AML: results of a phase 1 study. Blood. 2021;138(Supplement 1):2343–2343.

[140] Ruvolo PP, et al. Anexelekto/MER tyrosine kinase inhibitor ONO-7475 arrests growth and kills FMS-like tyrosine kinase 3-internal tandem duplication mutant acute myeloid leukemia cells by diverse mechanisms. Haematologica. 2017;102(12):2048–57. 28912176 10.3324/haematol.2017.168856 PMC5709104

[141] Lu JW, et al. Cabozantinib is selectively cytotoxic in acute myeloid leukemia cells with FLT3-internal tandem duplication (FLT3-ITD). Cancer Lett. 2016;376(2):218–25. 27060207 10.1016/j.canlet.2016.04.004

[142] Fathi AT, Blonquist TM, Hernandez D. *Cabozantinib is well tolerated in acute myeloid leukemia and effectively inhibits the resistance-conferring FLT3/tyrosine kinase domain/F691 mutation (vol 124, pg 306*, 2018). Cancer, 2018. 124(10): pp. 2258–2258. 10.1002/cncr.31038 PMC8167813 28960265

[143] Yan SB, et al. LY2801653 is an orally bioavailable multi-kinase inhibitor with potent activity against MET, MST1R, and other oncoproteins, and displays anti-tumor activities in mouse xenograft models. Investig New Drugs. 2013;31(4):833–44. 23275061 10.1007/s10637-012-9912-9 PMC3717159

[144] Kosciuczuk EM, et al. Merestinib blocks Mnk kinase activity in acute myeloid leukemia progenitors and exhibits antileukemic effects in vitro and in vivo. Blood. 2016;128(3):410–4. 27307295 10.1182/blood-2016-02-698704 PMC4957163

[145] Chen EC, et al. Targeting MET and FGFR in relapsed or refractory Acute myeloid leukemia: preclinical and clinical findings, and Signal Transduction Correlates. Clin Cancer Res. 2023;29(5):878–87. 36534523 10.1158/1078-0432.CCR-22-2540 PMC9992000

[146] Qian F, et al. Inhibition of tumor cell growth, invasion, and metastasis by EXEL-2880 (XL880, GSK1363089), a novel inhibitor of HGF and VEGF receptor tyrosine kinases. Cancer Res. 2009;69(20):8009–16. 19808973 10.1158/0008-5472.CAN-08-4889

[147] Wang P, et al. Foretinib is effective in Acute myeloid leukemia by inhibiting FLT3 and overcoming secondary mutations that Drive Resistance to Quizartinib and Gilteritinib. Cancer Res. 2024;84(6):905–18. 38231480 10.1158/0008-5472.CAN-23-1534 PMC10940854

[148] Eder JP, et al. A phase I study of foretinib, a multi-targeted inhibitor of c-Met and vascular endothelial growth factor receptor 2. Clin Cancer Res. 2010;16(13):3507–16. 20472683 10.1158/1078-0432.CCR-10-0574

[149] Patwardhan PP, et al. Significant blockade of multiple receptor tyrosine kinases by MGCD516 (Sitravatinib), a novel small molecule inhibitor, shows potent anti-tumor activity in preclinical models of sarcoma. Oncotarget. 2016;7(4):4093–109. 26675259 10.18632/oncotarget.6547 PMC4826192

[150] Zhang Y, et al. Sitravatinib as a potent FLT3 inhibitor can overcome gilteritinib resistance in acute myeloid leukemia. Biomark Res. 2023;11(1):8. 36691065 10.1186/s40364-022-00447-4 PMC9872318

[151] Chan WW, et al. Conformational control inhibition of the BCR-ABL1 tyrosine kinase, including the gatekeeper T315I mutant, by the switch-control inhibitor DCC-2036. Cancer Cell. 2011;19(4):556–68. 21481795 10.1016/j.ccr.2011.03.003 PMC3077923

[152] Cortes J, et al. Phase 1 dose-finding study of rebastinib (DCC-2036) in patients with relapsed chronic myeloid leukemia and acute myeloid leukemia. Haematologica. 2017;102(3):519–28. 27927766 10.3324/haematol.2016.152710 PMC5394958

[153] Hui Shi MW, Huang J, Ouyang Q, Guo J, Wang Y, Mi Y, Wu H. *CTS*2016, a novel AXL/FLT3 inhibitor for targeting AML/MDS and solid tumors, in *Proceedings of the American Association for Cancer Research Annual Meeting 2023*. 2023, AACR: Orlando, FL. p. Abstract nr 4021.

[154] Liu J, et al. Discovery of Novel small molecule mer kinase inhibitors for the treatment of Pediatric Acute Lymphoblastic Leukemia. ACS Med Chem Lett. 2012;3(2):129–34. 22662287 10.1021/ml200239k PMC3365829

[155] Christoph S, et al. UNC569, a novel small-molecule mer inhibitor with efficacy against acute lymphoblastic leukemia in vitro and in vivo. Mol Cancer Ther. 2013;12(11):2367–77. 23997116 10.1158/1535-7163.MCT-13-0040 PMC3823742

[156] Koda Y, Itoh M, Tohda S. Effects of MERTK inhibitors UNC569 and UNC1062 on the growth of Acute myeloid leukaemia cells. Anticancer Res. 2018;38(1):199–204. 29277773 10.21873/anticanres.12208

[157] Duan YT et al. A novel human anti-AXL monoclonal antibody attenuates tumour cell migration. Scand J Immunol, 2019. 90(2). 10.1111/sji.12777 31075180

[158] Duan Y, et al. Engineered AXL(-ECD)-Fc variants that abolish the AXL/Gas6 interaction suppress tumor cell migration. Oncol Lett. 2019;17(6):5784–92. 31186805 10.3892/ol.2019.10255 PMC6507473

[159] Liu Y, et al. Novel AXL-targeted agents overcome FLT3 inhibitor resistance in FLT3-ITD(+) acute myeloid leukemia cells. Oncol Lett. 2021;21(5):397. 33777220 10.3892/ol.2021.12658 PMC7988696

[160] Jin BK, et al. Nanobodies: a review of Generation, Diagnostics and therapeutics. Int J Mol Sci. 2023;24(6):5994. 36983063 10.3390/ijms24065994 PMC10057852

[161] Ackaert C, et al. Immunogenicity Risk Profile of Nanobodies. Front Immunol. 2021;12:632687. 33767701 10.3389/fimmu.2021.632687 PMC7985456

[162] Kariolis MS, et al. An engineered Axl ‘decoy receptor’ effectively silences the Gas6-Axl signaling axis. Nat Chem Biol. 2014;10(11):977–83. 25242553 10.1038/nchembio.1636 PMC4372605

[163] Kariolis MS, et al. Inhibition of the GAS6/AXL pathway augments the efficacy of chemotherapies. J Clin Invest. 2017;127(1):183–98. 27893463 10.1172/JCI85610 PMC5199716

